# Anserine reduces mortality in experimental sepsis by preventing methylglyoxal-induced capillary leakage

**DOI:** 10.1016/j.ebiom.2025.105644

**Published:** 2025-03-18

**Authors:** Thomas Schmoch, Nadia Gallenstein, Verena Peters, Maria Bartosova, Florian Uhle, Laura Kummer, Anian Mair, Ute Krauser, Manuel Feisst, Peter P. Nawroth, Markus A. Weigand, Claus Peter Schmitt, Thorsten Brenner

**Affiliations:** a Department of Anesthesiology and Intensive Care Medicine, University Hospital Essen, University Duisburg-Essen, Essen, Germany; b Medical Faculty Heidelberg, Department of Anesthesiology, Heidelberg University, Heidelberg, Germany; c Department of Anesthesiology and Intensive Care Medicine, Hôpitaux Robert Schuman – Hôpital Kirchberg, Luxembourg City, Luxembourg; d Medical Faculty Heidelberg, Department of Pediatrics I, Center for Paediatric and Adolescent Medicine, Heidelberg University, Heidelberg, Germany; e Institute of Medical Biometry, Heidelberg University, Heidelberg, Germany; f Medical Faculty Heidelberg, Department of Medicine I and Clinical Chemistry, Heidelberg University, Heidelberg, Germany

**Keywords:** Septic shock, Carbonyl stress, Anserine, Receptor of advanced glycation end products (RAGE)

## Abstract

**Background:**

We previously identified methylglyoxal as a biomarker for early identification and outcome prediction in human sepsis. We hypothesised that methylglyoxal causally impacts disease severity, and the methylglyoxal-scavenging dipeptide anserine can attenuate the detrimental effects of methylglyoxal.

**Methods:**

Using a translational approach, secondary analyses of two observational trials were performed to test the initial hypotheses. Afterwards, these results were re-evaluated in different murine models of experimental sepsis *in vivo*. The detrimental effects of methylglyoxal as well as the underlying mechanisms were further assessed *in vitro* using transendothelial electrical resistance measurements, fluorescence-activated cell sorting analyses, cytokine assays, gene expression analyses, and enzyme activity assays, as well as immunofluorescence and immunohistochemistry staining.

**Findings:**

The secondary analyses confirmed methylglyoxal as an independent marker associated with increased mortality within the first 48 h after sepsis onset and high catecholamine and fluid requirements in the first 24 h after sepsis onset. In the sepsis models, methylglyoxal-derived carbonyl stress significantly contributed to the development of capillary leakage by disrupting endothelial barrier–forming proteins. Mechanistically, a pathway involving the receptor of advanced glycation end products and mitogen-activated protein kinase was identified. The methylglyoxal-scavenging dipeptide anserine (β-alanyl-N-methylhistidine) reduced methylglyoxal-induced advanced glycation end-product formation and disruptions of junctional complexes *in vitro*. Moreover, anserine reduced capillary leakage and mortality *in vivo*.

**Interpretation:**

Methylglyoxal causally contributes to capillary leak formation and mortality in experimental sepsis, which can be mitigated by anserine. Therefore, anserine represents an innovative therapeutic option for the treatment of septic shock.

**Funding:**

10.13039/501100001659German Research Foundation (grant number BR 4144/2-1).

**Research in context**

**Evidence before this study**

In an observational clinical study, methylglyoxal was found to be superior to conventional markers of inflammation (interleukin 6, C-reactive protein, and procalcitonin) for identifying patients with sepsis. In addition, plasma concentrations of methylglyoxal at sepsis onset were significantly higher in non-surviving patients with sepsis as compared to sepsis survivors, suggesting a potential causal impact on the course of sepsis. Interestingly, this causal impact has already been shown for chronic diseases such as diabetes mellitus or neurodegenerative diseases, characterised by long-lasting but only moderately increased plasma concentrations of methylglyoxal for months to years. In this context, the dipeptide anserine has already been shown to prevent and partially reverse methylglyoxal-induced organ damage.

**Added value of this study**

Within the translational bench-to-bedside-and-back approach presented here, a short-term but strongly increased methylglyoxal load causally impacted the course of sepsis by capillary leak formation, resulting in septic shock. Moreover, anserine could counteract methylglyoxal-induced detrimental effects, resulting in an improved outcome in experimental sepsis.

**Implications of all the available evidence**

Methylglyoxal appears to play a clinically relevant role in the development of septic shock. As anserine is metabolised in the human liver from carnosine, which is a freely available and well-tolerated dietary supplement in many countries, anserine could be a promising new therapeutic option for the treatment of sepsis.

## Introduction

Despite decades of engaged sepsis research, our understanding of the underlying pathophysiology remains vague.^1^ There are few effective therapeutic options, and sepsis-associated mortality remains unacceptably high.^1^ Current knowledge about sepsis pathophysiology suggests that a dysregulated host response dominated by the innate immune system in the initial sepsis phase determines the disease course. The resulting systemic inflammation is associated with increased production of reactive oxygen species, reactive nitrogen species, and reactive carbonyl species.^2^^,^^3^ Whether and how the latter causally contributes to the pathophysiology of sepsis is unknown. In the context of diabetes mellitus, the reactive carbonyl species methylglyoxal (MG) is causally associated with the occurrence of late complications such as nephropathy, retinopathy, or neuropathy.^4^ In this context, MG is involved in the pathogenesis of blood-brain and blood-retinal barrier lesions associated with the development of diabetic retinopathy and diabetes-induced cognitive impairments.^5^^,^^6^ However, it remains unclear whether MG has a causal effect on the disease course in the hyperacute setting of sepsis. In a prospective observational study, we demonstrated that methylglyoxal (MG) is an early, sensitive, and specific marker for the identification of patients suffering from sepsis or septic shock, which is superior to established markers of inflammation and infection such as procalcitonin, C-reactive protein, and interleukin-6.^7^ Moreover, MG proved to be an early predictor of mortality in sepsis.^7^ In this translational bedside-to-bench-and-back approach, we investigated the causal impact of MG on the course of the disease to identify novel therapeutic approaches.

## Methods

### Reagents and antibodies

In this work, only commercially available cell lines and antibodies that had been validated by the supplier were used. All materials used, as well as the companies from which they were obtained, are listed in Supplementary Information (SI) Table S1.

### MG measurements

The MG concentration in heparin plasma was assayed by the liquid chromatography–mass spectrometry/mass spectrometry method described by McLellan and Thornalley.^8^

### Patient samples

To evaluate the association of MG plasma concentrations with patient survival^7^ and the association of MG plasma concentrations with patient fluid balance, catecholamine requirements, sequential sepsis-related organ failure assessment scores, and lactate plasma concentrations,^9^ we performed secondary analyses of preexisting data.^7^^,^^9^ Both trial protocols were registered in the German Clinical Trials Register [DRKS00000505^10^ and DRKS00012446^11^].

In both studies, the sex of the patients was documented on the basis of the information provided in the hospital information system. This information is entered on the basis of the data contained in the ID card or passport. In Germany, every citizen can self-report their own sex. However, no information about nationality, race, or ethnicity, was recorded in the studies.

### Mouse models

Female C57BL/6J mice (10–12 weeks of age, 18–22 g/mouse) were purchased from Charles River. Female C57BL/6N RAGE−/− GLO+/+ mice and the corresponding wild type animals from the same breeding (10–12 weeks of age, 18–22 g/mouse) were provided by the laboratory of the Department of Medicine I and Clinical Chemistry at the Heidelberg University (Heidelberg, Germany). In all experiments only female mice were used. Female laboratory animals are more robust than their male counterparts when exposed to experimental sepsis, which allows for more precise sample size planning and the use of smaller numbers of animals.^12^ The caecal ligation and puncture (CLP) sepsis mouse model was performed as previously described under general anaesthesia.^13^ For general anaesthesia, 120 mg ketamine + 16 mg xylazine were diluted with 0.9% NaCl to 10 ml. 10 ml/kg = 0.1 ml/10 g were injected intraperitoneally. Post-operative pain therapy was carried out with subcutaneous buprenorphine (0.1 mg/kg body weight) every 8 h, administered directly after the stress monitoring. For subcutaneous administration of MG (50 μg/g/day or placebo (NaCl 0.9%)), osmotic pumps (Techn. Details: 0.5 μl/h; running time 7 days) were used. Before CLP, prefilled osmotic pumps were inserted subcutaneously in the paravertebral region of anesthetised healthy C57BL/6J mice and continuously released MG or NaCl 0.9%. To induce systemic inflammation, ultrapure lipopolysaccharide (LPS) was administered by a single intraperitoneal injection (2.5 μg/g). Anserine (Ans) therapy was used as pretreatment every 12 h for 3 days (72 h) before the induction of sepsis via CLP. From wound closure on, Ans was used as posttreatment (after CLP) every 8 h for a maximum of 2 days (48 h). The dosage was 1.500 mg/kg intraperitoneally. For vascular permeability measurement, 1% Evans Blue was injected into the tail vein.^14^ The euthanasia of the laboratory animals was carried out under general anaesthesia by cervical dislocation. Detailed information about the planning and realisation of the mouse experiments can be found in Supplementary Information Methods (M) 1 and Table S2.

### Cell culture experiments

Primary human umbilical vein endothelial cells (HUVECs) were cultured using a complete endothelial cell growth medium supplemented with 1% Penicillin/Streptomycin.

### Transendothelial electrical resistance (TER)

HUVECs were grown on Transwell membrane filters for at least 3 days for the experiments as described by Srinivasan et al. (2015).^15^ The cells were utilised in passage 2–5. They were expanded at a confluence of 80%. The experiments were carried out at full confluence.

### Paracellular permeability assay

Permeability assays were performed as described by Ruiz-Remolina et al. (2017).^16^

### Fluorescence-activated cell sorter analysis

Fluorochrome-conjugated antibodies with the following specificities were used according to the manufacturers’ instructions: phospho-c-Jun, phospho-Akt, and phospho-p65. Measurements were performed on a BD FACS lyric (BD Bioscience) and the data were analysed using BD FACS Suite (v5).

### Caspase activity assay

Caspase activity was measured using the Caspase-Glo® Inflammasome Assay in a plate-reading luminometer (Tecan).

### Cytokine arrays

Cytokine protein concentrations (depicted as fold change compared with controls) were measured with cytokine arrays according to the recommended protocol with a charge-coupled device camera (Fusion FX7).

### Lactate dehydrogenase activity assay

Lactate dehydrogenase activity was measured with the Pierce LDH Cytotoxicity Assay Kit according to the recommended protocol.

### Trypan blue staining

In situ trypan blue staining was performed as described by Perry et al.^17^ according to the standard protocol.

### Immunofluorescence staining

HUVECs were stained with ZO-1-647, Claudin 5-488, and 4′,6-diamidin-2-phenylindol. Image acquisition was performed with an Acquifier imaging machine (ACQUIFIER). Images were analysed for immunofluorescence intensity on selected membrane areas in greyscale.^18^

### Immunohistochemistry

3,3′-diaminobenzidine staining was performed as described by Falangola et al.^19^ The slides were analysed digitally with Aperio software (Leica, Wetzlar, Germany).

### Evans Blue extraction and quantification

Evans Blue (EB) extraction and quantification were performed as described by Radu et al.^14^

### Measurement of Ans concentrations

Concentrations were measured using HPLC as described previously.^20^

### Sepsis definitions

In this work, sepsis was defined according to the Third International Definitions For Sepsis And Septic Shock (Sepsis-3). Details on this and further definitions are provided in Supplementary Information M2.

### Ethics

In the context of this work, secondary analyses of two studies were carried out.

The first study was ‘The different isoforms of the receptor for advanced glycation end products as key points in the septic immune response’ (RAMMSES-Trial).^7^^,^^10^ The RAMMSES-Trial was a prospective, observational, non-interventional, monocentric study which was conducted between August 2009 and July 2010 at the surgical intensive care unit of the University Hospital of Heidelberg, Germany. In brief, the aim of this study was to characterise in detail the various isoforms of RAGE as new forms of receptors in human sepsis.^10^ Furthermore, investigations were carried out to determine the connection between oxidative stress, carbonyl stress, RAGE expression, and nuclear factor kappa-light-chain-enhancer of activated B cells (NF-κB) activation within the field of human sepsis.^10^ Before including the first patient, the trial protocol was registered in the German Clinical Trials Register (Identifier: DRKS00000505) and approved by the local review board of the Heidelberg Medical Faculty (Trial Code Number S123-2009).^10^ The trial was carried out according to the Declaration of Helsinki and written informed consent, including secondary analyses, was obtained from all study participants.^21^

The second study was the ‘Prediction of acute kidney injury with the need for renal replacement therapy by the use of cell cycle arrest biomarkers in patients with sepsis or septic shock’ (PredARRT-Sep) trial,^9^ a prospective, exploratory observational study conducted in two ICUs at Heidelberg University Hospital between May 2017 and July 2018. One hundred patients with sepsis or septic shock (according to Sepsis-3) were included. In brief, the PredARRT-Sep trial investigated whether the product of the two cell cycle arrest biomarkers could be used to predict sepsis-induced acute kidney injury requiring dialysis.^1^^,^^9^ All patients were treated according to the guidelines of the Surviving Sepsis Campaign (SSC) valid at the time.^22^ Before including the first patient, the study was approved by the institutional review board (Trial Code Number S-200/2017) and registered at the German Clinical Trials Register (ID: DRKS00012446).^11^ The trial was carried out according to the Declaration of Helsinki and written informed consent, including secondary analyses, was obtained from all study participants.^21^

All animal experiments were performed per the appropriate guidelines and following approval (of a detailed protocol) from the Animal Care and Use Committee at the Regierungspräsidium Karlsruhe (AZ 35-9185.81/G-134/10, AZ 35-9185.81/G-106/15, AZ 35-9185.81/G-72/18). In addition, the ARRIVE guidelines 2.0 were followed in this paper.^23^

### Statistics

Wherever appropriate, the data are presented using bar charts, box and whisker plots, or Kaplan–Meier plots. The Kolmogorov–Smirnov test was applied to check whether the data followed a normal distribution. The MG concentrations, TER, paracellular leakage, EB concentrations, Ans concentrations, relative AGE-DAB positivity, and immunofluorescence intensity of two groups at individual time points were compared using the Mann–Whitney U-test. The log-rank test and the Gehan–Breslow–Wilcoxon method were used to compare two survival distributions. For experiments including mice, more information on sample size determination, randomisation, blinding, inclusion/exclusion criteria can be found in Supplementary Information Methods (M) 1.

The data were analysed using Microsoft® Excel 2016 (Microsoft Corporation, Redmond, USA), SPSS Statistics version 24.0 (IBM Corp., Armonk, NY, USA), and GraphPad Prism (Version 9.0.2, GraphPad Software, San Diego California USA). A p-value <0.05 was considered statistically significant in a descriptive sense.

### Role of funders

The funders had no role in study design, data collection, data analyses, interpretation, or writing of report.

## Results

### Methylglyoxal impacts the course of the disease in sepsis

**Fig. 1:**
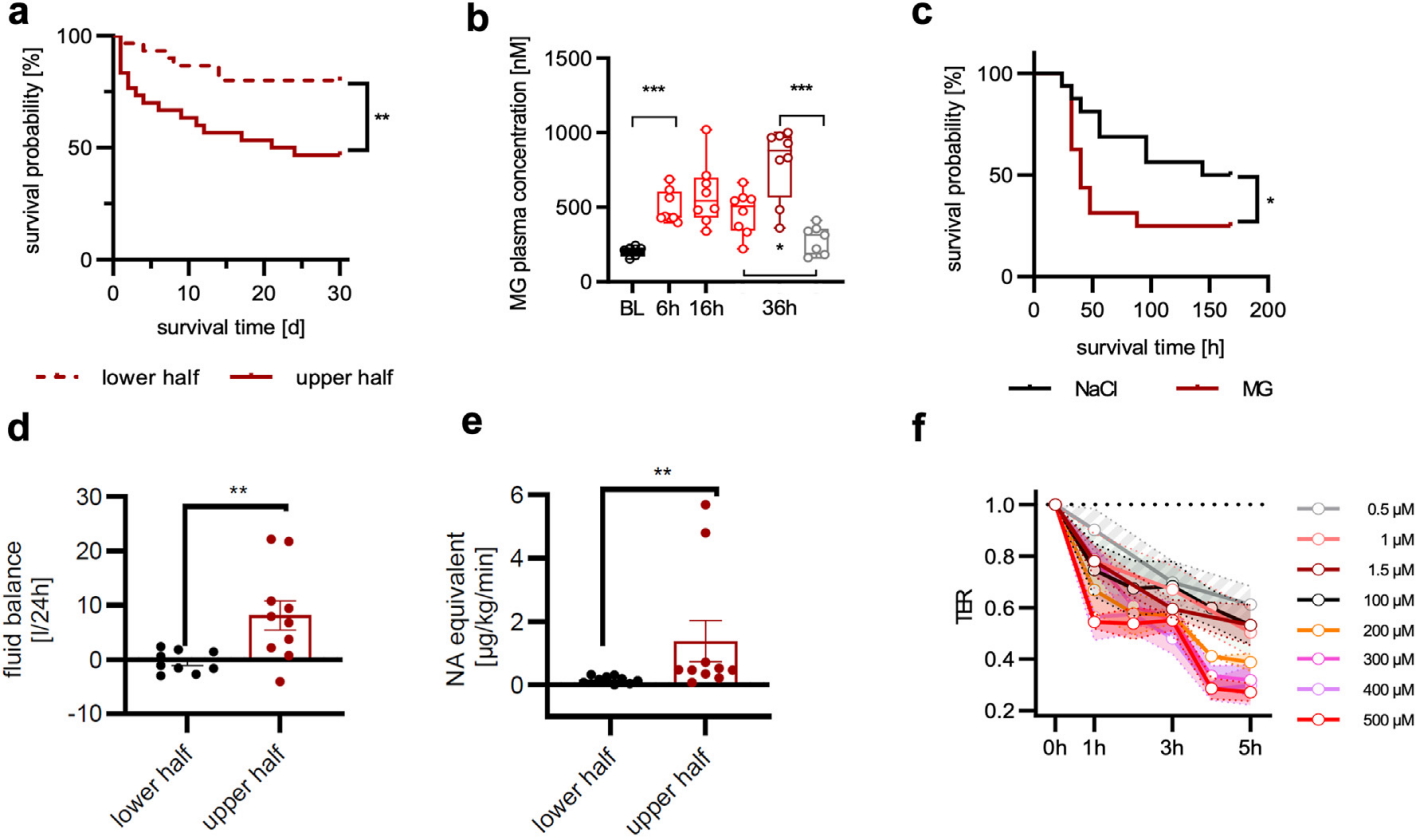
The plasmatic methylglyoxal load is increased in sepsis and directly impacts the outcome. a) Survival probability [%] of the 50% of patients with the highest MG plasma concentrations (=upper half), compared to the 50% of patients with lower MG plasma concentrations (=lower half). Secondary analysis from previously published data.^7^ n = 60; 30-day survival: p = 0.0064. Log-rank test. b) MG plasma concentrations in C57/BL6 mice 36 h following CLP (dark red box) or sham surgery (grey box), as well as 6, 16 and 36 h after LPS injection (2.5 mg/kg body weight/light red box) in comparison to BL controls (white box). Plasma concentrations of MG were measured using LC–MS/MS. Boxes visualise the median with the first to the third quartile; whiskers represent minimum and maximum. BL vs. LPS: 6 h p < 0.001; 36-h LPS vs. sham-surgery: p = 0.03 and sham-surgery vs. 36-h CLP: p ≤ 0.001. Log-rank test. c) Survival probability [%] of C57/BL6 mice receiving MG (50 μg/g/day, n = 16) vs. vehicle control (NaCl 0.9%; n = 16) following CLP. 168-h survival: p = 0.03. Gehan–Breslow–Wilcoxon test. d) Cumulative fluid balance, which is the sum of all infused fluids (crystalloids, colloids, blood products [tube] feedings, etc.) minus the sum of all fluid losses (urine, blood, perspiration insensibilis, etc.) (p = 0.008), and e) mean NA (equivalent) administration (p = 0.002) within the first 24 h after ICU admission (= sepsis onset) in the 50% of patients (n = 10) with the highest MG plasma concentrations (=upper half) vs. the 50% of the patients (n = 10) with lower MG plasma concentrations (=lower half).^9^ For details, see SI Table S2A. n = 20 random samples out of n = 100 included patients. For data comparing the random samples to the entire cohort, see SI Table S2B. Data are presented as mean and SEM. Mann–Whitney U-test. f) Time course of TER of HUVEC monolayers under MG treatment relative to media control. Treatment was repeated every 2 h. Data are presented as the mean and SEM of n = 6. For supportive statistics see SI Table S3. Symbols: ∗p ≤ 0.05; ∗∗p ≤ 0.01; ∗∗∗p ≤ 0.001. Abbreviations: BL, baseline; CLP, caecal ligation and puncture; HUVEC, human umbilical vein endothelial cells; ICU, intensive care unit; LC–MS/MS, liquid chromatography-mass spectrometry/mass spectrometry; LPS, lipopolysaccharide; MG, methylglyoxal; NA, noradrenaline; SEM; standard error of the mean; TER, transendothelial electrical resistance.

In a secondary analysis of our above-mentioned observational study,^7^ the 50% of patients with sepsis with the highest MG plasma concentrations at the time of sepsis diagnosis (here referred to as the ‘upper half’) were compared to the 50% of patients with the lowest MG plasma concentrations (the ‘lower half’) (SI Table S3; SI Figure S1a–c).^7^ The upper half had a significantly higher 48-h mortality (7/30 [23.3%] vs. 1/30 [3.3%], hazard ratio [HR] = 7.3 [95% confidence interval (CI) 1.8–29.1], p = 0.02, log-rank test) and 30-day mortality (16/30 [53.3%] vs. 6/30 [20.0%], HR = 3.3 [95% CI 1.4–7.8], p = 0.0064, log-rank test) compared to patients with lower MG plasma concentrations (Fig. 1a).

To determine whether MG is merely a surrogate parameter for a severe disease course or whether it causally contributes to mortality in sepsis, we next transferred our human findings to a mouse model of experimental sepsis. After CLP and after LPS injection, mice developed plasmatic MG kinetics comparable to those observed in early human sepsis (Fig. 1b).^7^ Moreover, an artificial elevation of MG stress significantly increased mortality, especially within the first 48 h after sepsis induction (48-h mortality: 11/16 [68.8%] vs. 3/16 [18.8%], HR = 4.4 [95% CI 1.5–12.6], p = 0.008, Gehan–Breslow–Wilcoxon method; 168-h mortality: 12/16 [75.0%] vs. 8/16 [50.0%], HR = 2.2 [95% CI 0.9–5.4], p = 0.03, Gehan–Breslow–Wilcoxon method; Fig. 1c).

### MG impairs the endothelial barrier

As 48-h mortality was increased in patients with sepsis with high MG plasma concentrations and septic mice receiving additional MG administration, we hypothesised that MG may causally contribute to the development of septic shock associated with a profound disturbance of the endothelial barrier.^24^^,^^25^ Seeking further arguments to support this hypothesis, we quantified MG plasma concentrations in another random sample of 20 patients with sepsis or septic shock on admission to the intensive care unit, which participated in a second observational clinical study (SI Table S4A and B)^9^ and compared the 50% of patients with the highest MG plasma concentrations (‘upper half’) to those with the lowest MG plasma concentrations (‘lower half’). We found that higher MG concentrations are associated with a significantly increased fluid load and a greater need for vasopressors within the first 24 h after sepsis onset (Fig. 1d and e); however, no differences were detected regarding Sequential Sepsis-related Organ Failure Assessment scores and plasmatic lactate concentrations (SI Figure S1e and f).

To further assess the potential impact of MG on endothelial barrier function, we used primary human umbilical vein endothelial cells (HUVECs). To evaluate endothelial integrity, we measured the TER of HUVEC monolayers exposed to MG over a 5-h time course. The MG concentration in the cell culture medium was adjusted every 2 h. In this experimental setup, MG reduced the TER of HUVEC monolayers in a dose-dependent manner (starting from levels observed in patients with sepsis; 0.5–1.5 μM) and depending on the exposure time (Fig. 1f; SI Table S5). Similar reductions in TER were observed in HUVEC monolayers exposed to LPS and tumour necrosis factor (TNF), respectively (SI Figure S1g and h; SI Table S5). Since MG (at the levels observed in sepsis patients) was able to disrupt the TER *in vitro* as significantly as LPS or TNF, two molecules well known to induce capillary leakage in sepsis, we concluded that MG could be a crucial factor in the induction of capillary leakage leading to septic shock.^26^

### MG effects in sepsis are RAGE/mitogen-activated protein kinase mediated

**Fig. 2:**
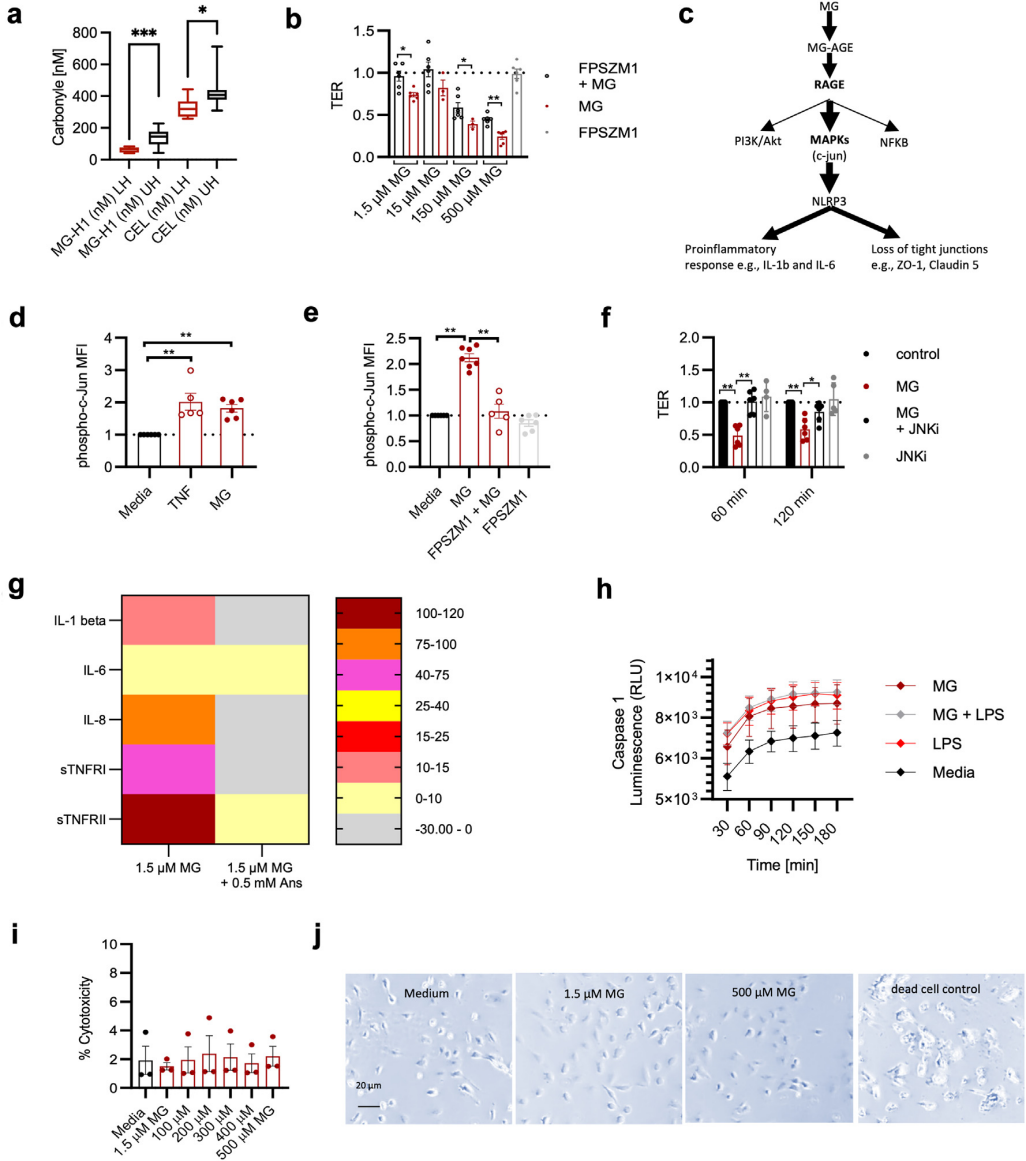
MG induces inflammation. a) MG-associated hydroimidazolone (MG-H1) and N(6)-(carboxyethyl)lysine (CEL) plasma concentrations at sepsis onset in the 50% of patients (n = 10) with the highest MG plasma concentrations (the upper half, UH) versus the 50% of the patients (n = 10) with lower MG plasma concentrations (the lower half, LH). b) The 5-h TER ratios following a repetitive treatment with MG alone or MG with FPSZM1 (1 μM, pretreated for 2 h), normalised to media control. 1.5 μM MG vs. 1.5 μM MG + FPSZM1: p = 0.0173; 150 μM MG vs. 150 μM MG + FPSZM1: p = 0.0238; 500 μM MG vs. 500 μM MG + FPSZM1: p = 0.0022. Data are presented as mean and SEM. Mann–Whitney U-test. c) Scheme of the proposed pathway. d) Phospho-c-Jun FACS staining in HUVECs after single treatments with 150 μM MG or TNF (50 ng/ml) for 30 min normalised to media control. Media vs. TNF: p = 0.0022; Media vs. MG: p = 0.002. Data represent mean and SEM. Mann–Whitney U-test. e) MFI quotients for phospho-c-Jun after 2 h FPSZM1 (1 μM) pretreatment and/or a single dose of 150 μM MG for 30 min normalised to media control. Media vs. MG: p = 0.0012; FPSZM1 + MG vs. MG: p = 0.0025. Data represent mean and SEM. Mann–Whitney U-test. f) TER ratios following a single treatment with MG (150 μM) alone or MG in combination with the c-Jun inhibitor SP600125 (50 μM, pretreated for 20 h) and JNKi control, normalised to media control. At 60 min: control vs. MG: p = 0.0022; MG vs. MG JNKi: p = 0.002; at 120 min: control vs. MG: p = 0.002; MG vs. MG JNKi: p = 0.0238. Data represent mean and SEM. Mann–Whitney U-test. g) Time course of caspase-1 activity in HUVECs, treated for 2 h with a pathophysiological MG dose (1.5 μM) and/or LPS (1 μg/ml). Data are presented as mean and SEM. h) Inflammatory protein profile excreted from a HUVEC monolayer after incubation with MG only or MG and Ans for 5 h in comparison with MG-free media control (Cytokine array). MG was administered every 2 h, and Ans was administered once together with the first MG treatment. Heat map represents protein concentrations measured by dot intensity differences between treatment and control values of batched supernatants from n = 4 experiments. i) LDH cytotoxicity assay. HUVECs were treated with different MG concentrations for 5 h. Data are presented as mean and SEM. j) Representative images of in situ trypan blue cell viability staining of repeatedly MG-treated HUVECs and medium control after 5 h. Dead cell control: 100% EtOH. Symbols: ∗p ≤ 0.05; ∗∗p ≤ 0.01. Abbreviations: Ans, anserine; c-Jun, c-Jun N-terminal kinase; FACS, fluorescence-activated cell sorting; HUVEC, human umbilical vein endothelial cells; IL, interleukin; JNKi, c-Jun N-terminal kinase inhibitor; LDH, lactate dehydrogenase; LPS, lipopolysaccharide; MFI, mean fluorescence intensity; MG, methylglyoxal; RLU, Relative luminescence unit; SEM, standard error of the mean; sTNFR, Soluble tumour necrosis factor receptors; TER, transendothelial electrical resistance; TNF, tumour necrosis factor.

MG binds to proteins (e.g. albumin, collagen, and enzymes), deoxyribonucleic acid and phospholipids, altering their structure and impairing or changing their original function. These modified molecules are called MG-advanced glycation end products (AGEs). The MG-AGEs, along with other AGEs and many other proteins (e.g. high-mobility group box 1 [HMGB1], and S100 proteins), can bind to RAGE, which is known to contribute to acute and chronic inflammation.^27^ The two most important MG-related AGEs are MG-associated hydroimidazolone (MG-H1) and N(6)-(carboxyethyl)lysine (CEL).^4^^,^^28^, ^29^, ^30^, ^31^ In the above-mentioned random samples from an observational study, the 50% of the patients with the highest MG plasma concentrations (‘upper half’) also had significantly increased MG-H1 and CEL plasma concentrations (Fig. 2a).^9^ To distinguish whether the observed MG effects in sepsis were caused by direct toxicity or predominantly mediated by RAGE, we used the specific RAGE-blocking agent FPS-ZM1. This prevented MG-induced loss of TER *in vitro* (Fig. 2b; SI Figure S2a and b). After its activation, RAGE acts via three different downstream pathways. The first is IκB kinase signalosome-dependent phosphorylation of NF-κB inhibitors (IκB inhibitors) and thus activation of the NF-κB pathway, which subsequently induces the synthesis of proinflammatory cytokines.^32^ The second is the phosphoinositide 3-kinase/protein kinase B pathway and the third is the activation of mitogen-activated protein (MAP) kinase members, including the Jun-N-terminal kinase, p38, and the extracellular signal-regulated kinase (Fig. 2c).^33^ Fluorescence-activated cell sorting (FACS) analysis revealed the activation and respective phosphorylation of the Jun-N-terminal kinase ‘c-Jun’, but not the p65 subunit of NF-κB or protein Kinase B (Akt), following MG exposure (Fig. 2d; SI Figure S2c and d). In line with this, the blockade of RAGE with FPS-ZM1 prevented c-Jun phosphorylation (Fig. 2e), and inhibition of c-Jun phosphorylation prevented TER loss (Fig. 2f; SI Figure S2e). To determine which downstream inflammatory processes could be induced by MG, we used a cytokine array to examine the expression changes in 60 interleukins. We found that MG exposure in HUVECs induces interleukins typical of the early phase of sepsis (e.g. interleukin-6, 8, and 1β, as well as soluble TNF receptor I and II, at the time of TER loss [Fig. 1f]), as they are pro-inflammatory and activate the innate immune response (Fig. 2g, first column; SI Figure S3, first column).^26^

The presence of IL-1β indicates the activation of the nucleotide-binding oligomerisation domain (NOD) leucine-rich repeat (LRR)-containing protein (NLR) family member (NLRP-3) inflammasome, which was verified by the detection of caspase-1 following MG stimulation (Fig. 2h). In line with these findings, inhibition of the NLRP-3 inflammasome assembly prevented MG-induced loss of TER (SI Figure 2f). Of note, our findings cannot be attributed to an increase in pyroptosis, as the loss of TER was not associated with increased cell death (Fig. 2i and j).

**Fig. 3:**
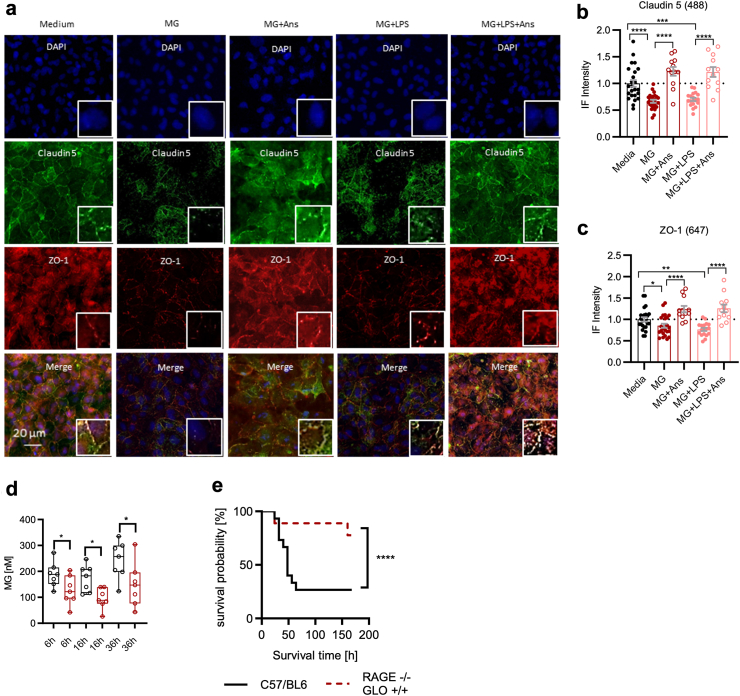
MG impairs tight junction function and survival in sepsis. a) Representative immune fluorescence claudin 5 and ZO-1 staining in HUVECs treated (from left to right) with media, 1.5 μM MG, 1.5 μM MG + 0.5 mM Ans, 1.5 μM MG + 1 μg/ml LPS, or 1.5 μM MG + 1 μg/ml LPS + 0.5 mM Ans for 5 h. MG was administered every 2 h. b and c) Bar graphs of relative IF intensity of HUVEC stained for claudin 5 (b) and ZO-1 (c). HUVEC were treated as described in a) for 5 h. MG was administered every 2 h. Claudin 5: control vs. MG: p ≤ 0.0001; control vs. MG + LPS: p = 0.003; MG vs. MG + Ans: p ≤ 0.0001; MG + LPS vs. MG + LPS + Ans: p ≤ 0.0001. ZO-1: control vs. MG: p = 0.0298; control vs. MG + LPS: p = 0.0011; MG vs. MG + Ans: p ≤ 0.0001; MG + LPS vs. MG + LPS + Ans: p ≤ 0.0001. Each bar represents the mean IF intensity relative to media control. Data are presented as mean and SEM. Mann–Whitney U-test. d) MG plasma concentrations in septic C57/BL6 RAGE−/+ GLO-1+/− (black) or RAGE−/− GLO-1+/+ (dark red) mice at 6 h (p = 0.05), 16 h (p = 0.02), and 36 h (p = 0.05) following CLP (n = 7 per group and time point). MG plasma concentrations were measured by LC-MS/MS. Boxes visualise the median with the first to the third quartile; whiskers represent the minimum and maximum. Mann–Whitney U-test. e) Survival probability [%] of RAGE−/− GLO+/+ mice (n = 9) and C57/BL6 (n = 15) after induction of experimental sepsis via CLP. Survival: wild-type C57/BL6 mice 6/15 [42.9%] vs. RAGE−/− GLO+/+ mice 7/9 [77.8%]; HR = 0.2 [95% CI 0.07–0.65], p ≤ 0.03. Gehan–Breslow–Wilcoxon test. Symbols: ∗p ≤ 0.05; ∗∗p ≤ 0.01; ∗∗∗∗p ≤ 0.0001. Abbreviations: Ans, anserine; CLP, caecal ligation and puncture; GLO1, glyoxalase 1; HUVEC, human umbilical vein endothelial cells; IF, immune fluorescence; LC–MS/MS, liquid chromatography–mass-spectrometry/mass spectrometry; LPS, lipopolysaccharide; MG, methylglyoxal; RAGE, receptor of advanced glycation end products; SEM, standard error of the mean; TER, transendothelial electrical resistance.

Our findings correspond to a rarefication of tight junction proteins on a histopathological level (tight junction-associated protein 1 [ZO-1] and claudin 5) (Fig. 3a–c, columns 1, 2 and 4). On the one hand, we were able to demonstrate that (1) RAGE inhibition can reduce the effects of MG on TER and that MG induces (2) phosphorylation of c-Jun as well as (3) caspase-1. However, the inhibition of c-Jun phosphorylation can (4) prevent the induction of caspase-1 in this model and (5) prevent TER loss. To test *in vivo* whether the reduction of MG stress to a minimum would substantially improve the clinical situation, we used a RAGE-knock out (KO−/−) mouse strain with increased expression of the MG-detoxifying enzyme glyoxalase-1 (due to gene doubling),^34^ reducing both the direct toxicity and the RAGE-mediated effects of MG. Indeed, the RAGE−/− GLO+/+ genotype was associated with significantly lower MG plasma concentrations in experimental sepsis (Fig. 3d) as well as a significantly increased survival rate in comparison with wild-type mice (survival: wild-type C57/BL6 mice 6/15 [42.9%] vs. RAGE−/− GLO+/+ mice 7/9 [77.8%]; HR = 0.2 [95% CI 0.07–0.65], p ≤ 0.03, Gehan–Breslow–Wilcoxon method; Fig. 3e).

### Anserine improves survival in experimental sepsis

**Fig. 4:**
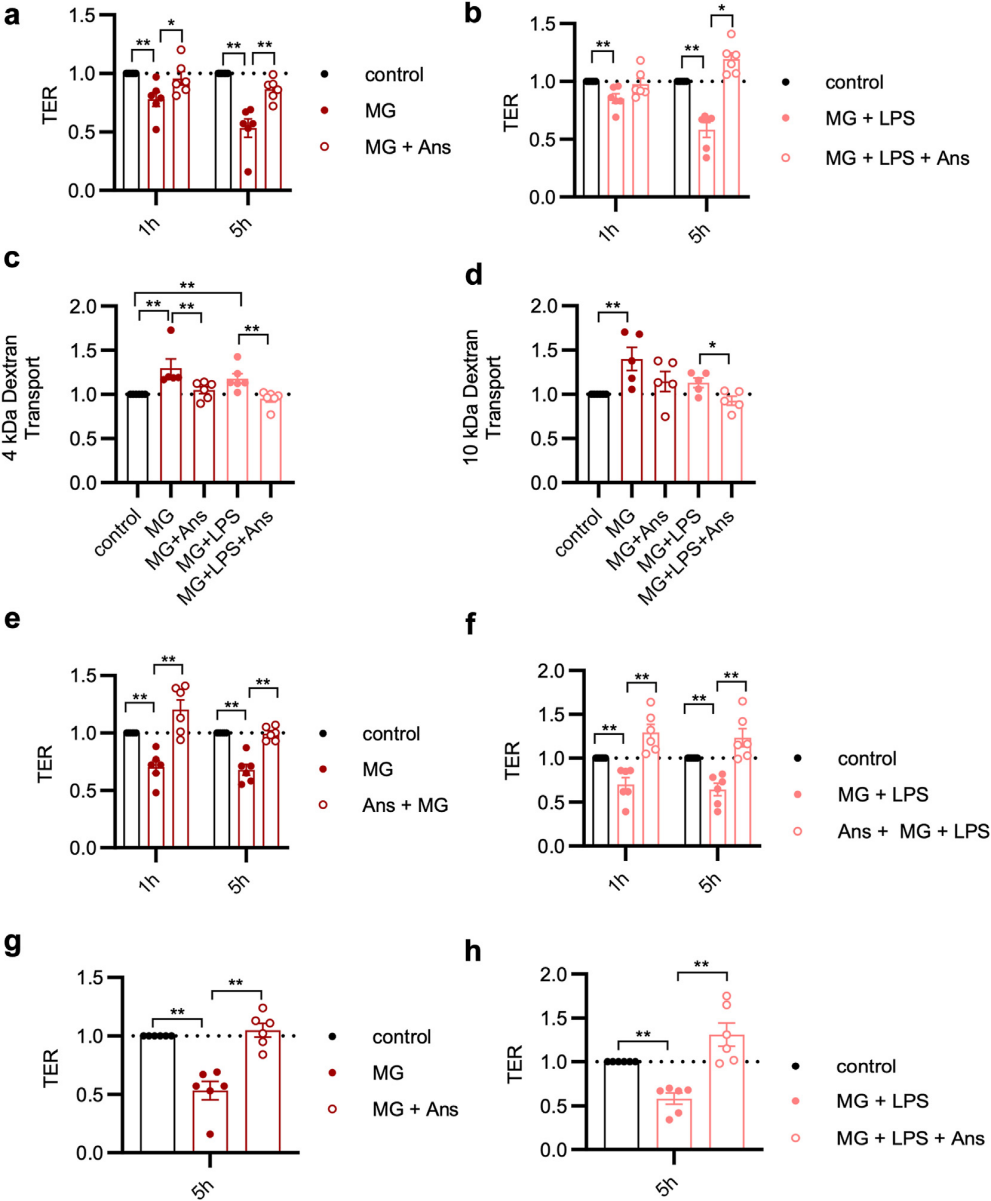
Ans prevents MG- and LPS-induced impairments of the endothelial barrier. a) Effect of MG (1.5 μM) and Ans (0.5 mM) cotreatment on the TER of a HUVEC-polarised monolayer relative to untreated media control (1 h, control vs. MG: p = 0.002, MG vs. MG + Ans = 0.04; 5 h, control vs. MG: p = 0.002, MG vs. MG + Ans: p = 0.002). Mann–Whitney U-test. b) Effect of MG with concomitant LPS (1 μg/ml) stimulation and Ans cotreatment on the TER of a HUVEC polarised monolayer (1 h, control vs. MG + LPS: p = 0.002; 5 h, control vs. MG + LPS: p = 0.002, MG + LPS vs. MG + LPS + Ans: p = 0.01). Mann–Whitney U-test. c) Effect of 5 h MG exposure (1.5 μM; with or without a concomitant LPS stimulation (1 μg/ml)) and cotreatment with 0.5 mM Ans on the paracellular leak of 4 kDa dextran (control vs. MG: p = 0.002; control vs. MG + LPS: p = 0.002; MG vs. MG + Ans, p = 0.004; MG + LPS vs. MG + LPS + Ans, p = 0.002) through a primary HUVEC monolayer. Mann–Whitney U-test. d) Effect of 5 h of MG exposure (1.5 μM; with or without a concomitant LPS stimulation; 1 μg/ml) and cotreatment with 0.5 mM Ans on the paracellular leak of 10 kDa dextran (control vs. MG: p = 0.002; MG + LPS vs. MG + LPS+ Ans, p = 0.03) through a primary HUVEC monolayer. Mann–Whitney U-test. e) Protective effect of an Ans pretreatment followed by repeated MG (1 h, control vs. MG: p = 0.002, MG vs. MG + Ans: p = 0.002; 5 h, control vs. MG: p = 0.002, MG vs. MG + Ans: p = 0.002) or f) repeated MG and a single LPS (1 h, control vs. MG + LPS: p = 0.002, MG + LPS vs. MG + LPS + Ans: p = 0.002; 5 h, control vs. MG + LPS: p = 0.002, MG + LPS vs. MG + LPS + Ans: p = 0.002) administration on a primary HUVEC monolayer. Mann–Whitney U-test. g) Rescue effect of Ans on an MG-exposed primary HUVEC monolayer. After 3 h of MG exposure (control vs. MG: p = 0.002, MG vs. MG + Ans: p = 0.002) or h) MG and LPS exposure (control vs. MG + LPS: p = 0.002, MG + LPS vs. MG + LPS +Ans: p = 0.002), a single dose of Ans was given. Mann–Whitney U-test. Symbols: ∗p ≤ 0.05; ∗∗p ≤ 0.01. Plotted data represent mean and SEM. Abbreviations: Ans, anserine; HUVEC, human umbilical vein endothelial cells; LPS, lipopolysaccharide; MG, methylglyoxal; SEM, standard error of the mean; TER, transendothelial electrical resistance.

The dipeptides carnosine (Cns) and anserine (Ans) mitigate diabetic late complications *in vitro* and *in vivo*^35^^,^^36^ and dose-dependently reduce the formation of MG-derived AGEs.^37^ Therefore, we hypothesised that both dipeptides could reduce the MG-induced impairment of the endothelial barrier in sepsis. Indeed, Ans prevented an MG-induced as well as an LPS-induced loss of TER (Fig. 4a and b). Correspondingly, Ans prevented the MG- and LPS-induced increase in the paracellular leakage of the 4 kDa, 10 kDa (Fig. 4c and d) and 70 kDa dextran (SI Figure S4a). The TER-protective effects remained significant when incubating HUVECs with supraphysiological MG doses of up to 500 μM as long as Ans concentrations were increased proportionally (SI Figure S4b and c). The maintenance of TER was associated with the preservation of the tight junctions (Fig. 3a and b). Moreover, Ans treatment prevented the increase in proinflammatory cytokines (e.g. IL-6 and IL-1β) in HUVECs exposed to MG. Fig. 2g and SI Figure S3 (second column) provide an overview of the cytokines studied, covering the majority of those relevant for sepsis.^38^

Like Ans, Cns prevented a loss of TER 1 h following exposure to MG and LPS. However, 4 h later this effect was no longer significant (SI Figure S4d and e). Therefore, our work continued to focus on Ans. Of note, the protective effects of Ans were comparable to those of aminoguanidine (AG), a well-characterised *in vitro* MG scavenger (SI Figure S4f and g; SI Figure S5a and b).^39^, ^40^, ^41^ To exclude the possibility that a simple antioxidant effect could explain the protective effects of Ans, we treated MG-stressed endothelial cells with N-acetyl-cysteine. However, N-acetyl-cysteine treatment did not prevent an MG-induced loss of TER (SI Figure S4h and i; SI Figure S5a and c). Therefore, we hypothesise that the capillary leakage observed in sepsis is caused by reactive carbonyl species rather than reactive oxygen species stress.

Although the formation of MG-AGE is a relatively slow process, we wondered whether a prophylactic application of Ans could further enhance its protective effect.^42^ Starting Ans treatment 2 h before the first MG exposure had comparable TER-preserving effects (Fig. 4e and f) as compared to the cotreatment (MG exposure and Ans treatment starting simultaneously, Fig. 4a and b). Next, we investigated whether Ans was able to restore endothelial integrity when the treatment started after endothelial MG had already been established. A single dose of Ans added 3 h after the start of MG and MG + LPS exposure also restored the loss of TER (Fig. 4g and h).

**Fig. 5:**
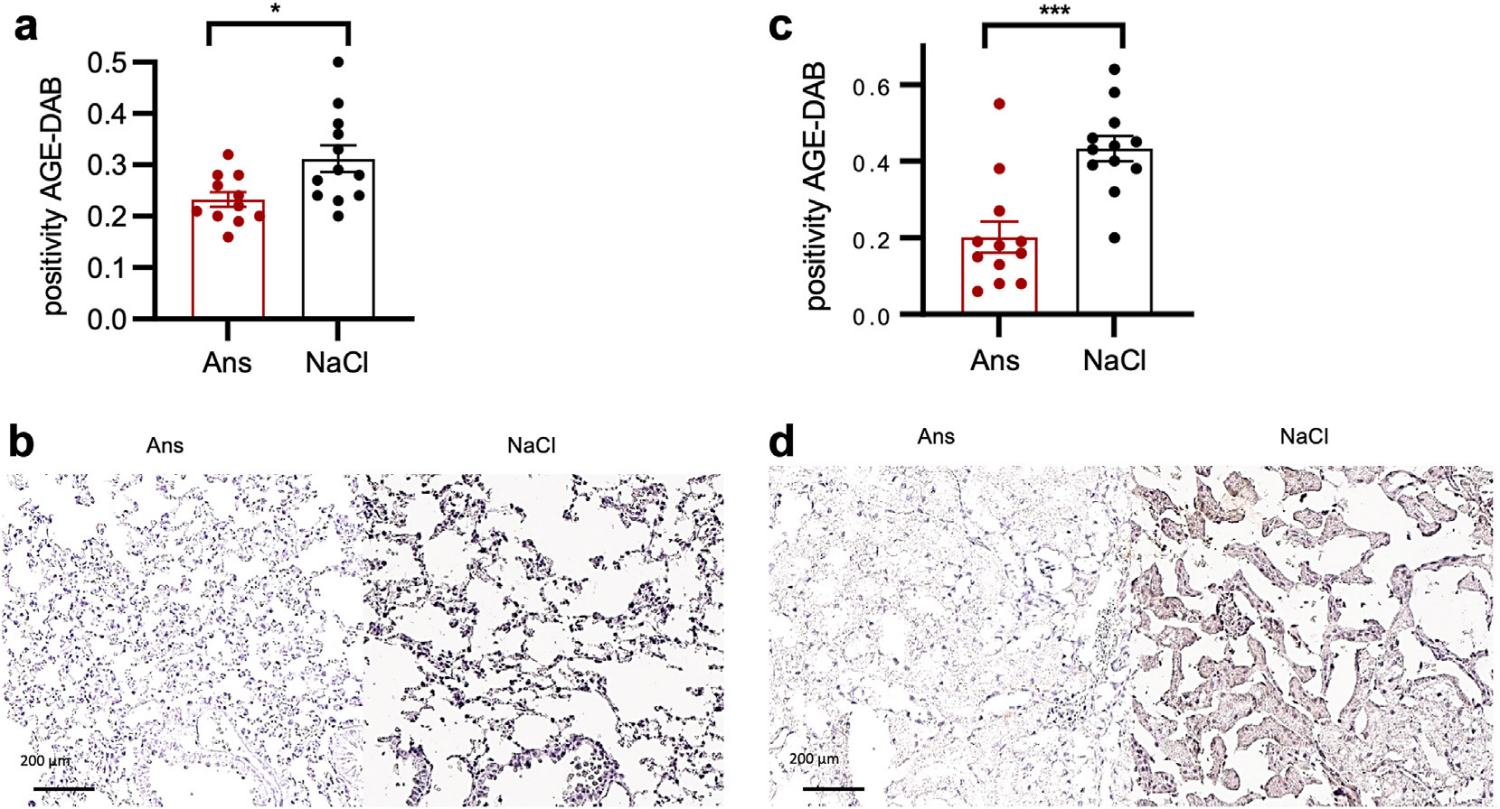
Ans antagonises the MG effect *in vivo*. a) AGE distribution in septic mice at 24 h following CLP under Ans treatment vs. vehicle control. Immunohistochemical quantification of lung tissue (n = 6/group, p = 0.01; data are represented as mean and SEM. Mann–Whitney U-test), and b) representative images of AGE–3,3′-diaminobenzidine staining of lungs. c) AGE distribution in septic mice 24 h after CLP under Ans treatment vs. vehicle control. Immunohistochemical quantification of kidney tissue (n = 6/group, p = 0.0003; data represent mean and SEM. Mann–Whitney U-test), and d) representative images of AGE–3,3′-diaminobenzidine staining of kidneys. Symbols: ∗p ≤ 0.05; ∗∗∗p ≤ 0.001. Abbreviations: AGE, advanced glycation end product; Ans, anserine; CLP, caecal ligation and puncture; DAB, 3,3′-diaminobenzidine; MG, methylglyoxal; SEM, standard error of the mean.

As a proof of concept, we then retranslated the aforementioned *in vitro* findings back into the CLP mouse model of sepsis, in which Ans treatment reduced AGE concentrations in the stroma of the lungs and kidneys (Fig. 5a–d).

**Fig. 6:**
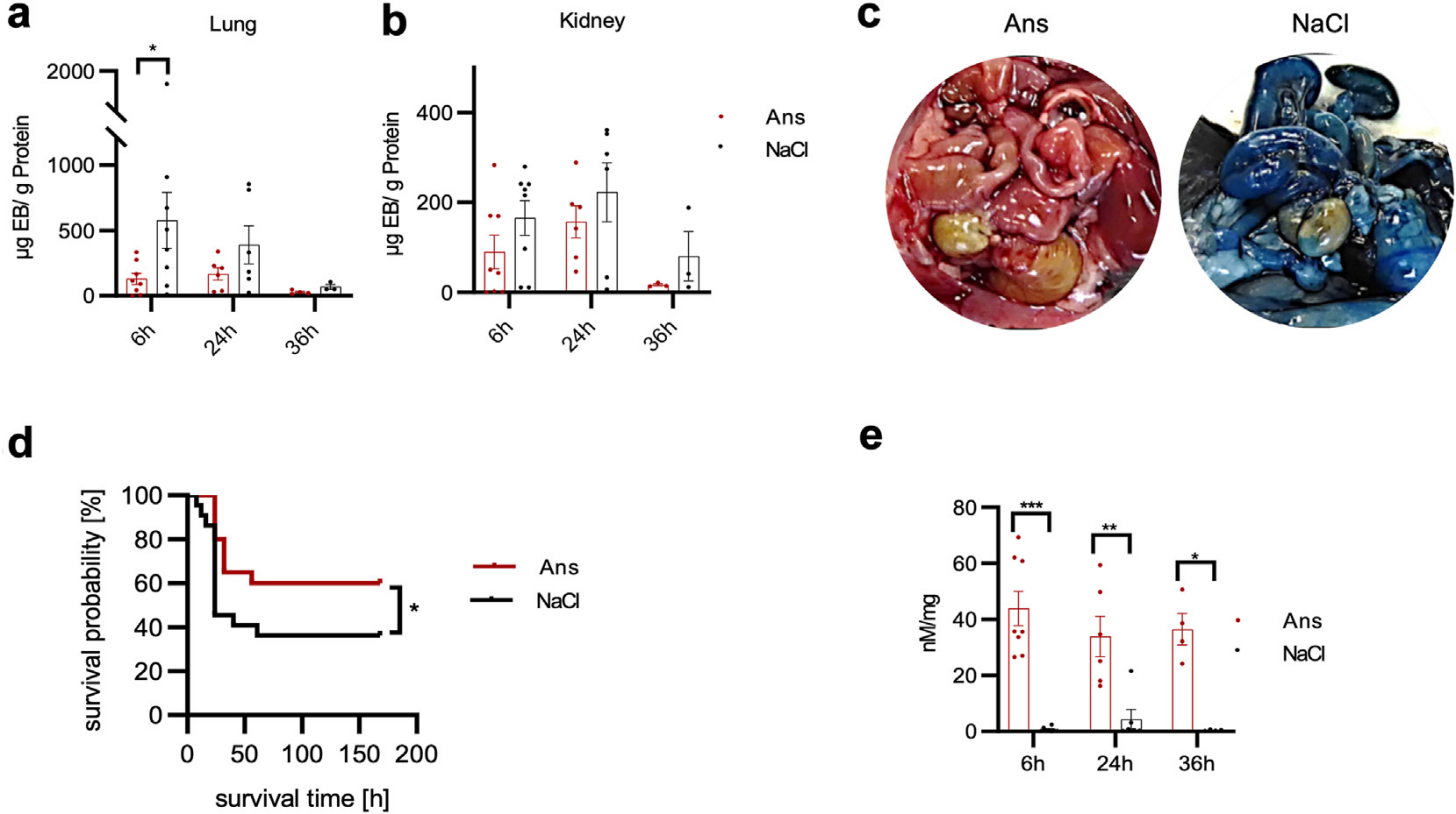
Ans improves survival in septic mice. a) Summary of EB extravasation in lungs of septic mice at 6 h (p = 0.04), 24 h and 36 h after CLP under Ans treatment vs. vehicle control. Data are represented as mean and SEM. Mann–Whitney U-test. b) Summary of EB extravasation in kidneys of septic mice at 6 h, 24 h and 36 h after CLP under Ans treatment vs. vehicle control. Data are represented as mean and SEM. Mann–Whitney U-test. c) Representative images of septic mice following CLP under Ans treatment vs. vehicle control, injected with EB (1%) at 24 h. d) Survival probability [%] of septic mice up to 168 h following CLP, which were pre- and post-treated with vehicle (n = 22) or Ans (n = 20): vehicle 6/22 [27.3%] vs. Ans 12/20 [60.0%]; HR = 0.5 (CI 0.2–1.2), p = 0.04. Gehan–Breslow–Wilcoxon test. e) Ans concentrations at 6 h (n = 8, p < 0.001), 24 h (n = 6, p = 0.009), and 36 h (n = 4, p = 0.02) in septic mice following CLP under Ans treatment vs. vehicle control in kidney tissue measured by HPLC. Data represent mean and SEM. Mann-Whitney-U test. Concerning symbols: ∗p ≤ 0.05; ∗∗p ≤ 0.01; ∗∗∗p ≤ 0.001. Abbreviations: Ans, anserine; EB, Evans blue; HPLC, high-performance liquid chromatography; CLP, caecal ligation and puncture; MG, methylglyoxal; SEM, standard error of the mean.

Moreover, Ans treatment significantly reduced capillary leakage (Fig. 6a–c; SI Figure 6a and b) and seven-day mortality (40.0% vs. 63.6%; p = 0.04, Gehan–Breslow–Wilcoxon method) in CLP-induced experimental sepsis in mice (Fig. 6d). In treated tissues, Ans was stably increased over 36 h (kidneys Fig. 6e; heart and lungs SI Figure S7a and b). In addition, there was a positive correlation of the Ans concentration in the plasma and the kidney, as well as in the plasma and the heart after 24 h (SI Figure S7c).

**Fig. 7:**
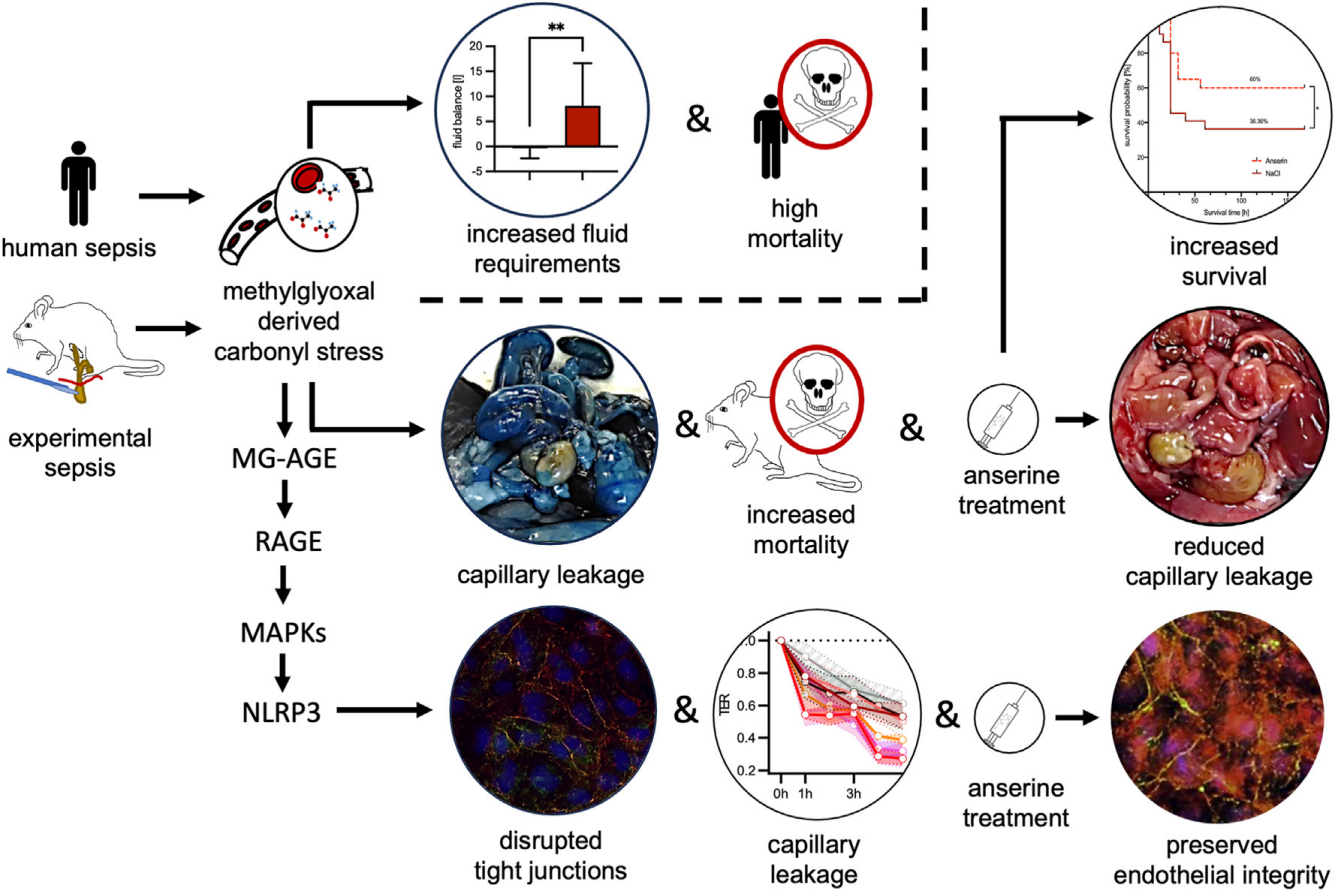
Graphical abstract. Abbreviations: AGEs, advanced glycation end products; MAPK, mitogen-activated protein kinase; MG, methylglyoxal; NLRP3, nucleotide-binding oligomerisation domain (NOD) leucine-rich repeat (LRR)-containing protein (NLR) family member 3.

Taken together, in a secondary analysis of an observational clinical study we found that high MG plasma concentrations were associated with early death in sepsis patients (within 48 h after sepsis onset; Fig. 1a). At the same time, high MG plasma concentrations were associated with a positive fluid balance and an increased catecholamine requirement, making the association with septic shock even more plausible (Fig. 1d and e). In our *in vitro* experiments, MG increased endothelial permeability (Fig. 1f) and there was a loss of tight junction proteins (Fig. 3a–c). *In vivo*, such an effect of MG could favour the development of a capillary leak, a hallmark of septic shock. Mechanistically, the observed MG-associated effects were neither exclusively non-specific cytotoxic effects (Fig. 2i and j) nor due to oxidative stress (SI Figure S4h and i; SI Figure S5a and c). Instead, the loss of the endothelial cell barrier could be significantly reduced with the specific RAGE blocker FPS-ZM1 (Fig. 2b). Downstream, the loss of endothelial barrier function was associated with phosphorylation (activation) of c-Jun (which could also be prevented by the RAGE blocker FPS-ZM1; Fig. 2d and e). Conversely, inhibition of this phosphorylation prevented loss of the endothelial barrier (Fig. 2f). Furthermore, we found strong evidence that caspase-1 and the NLRP-3 inflammasome are involved in the pathophysiology further downstream. First, MG induced caspase-1, which is characteristic of this inflammasome (Fig. 2h). Second, the inhibition of inflammasome assembly prevented MG-induced loss of the endothelial barrier (SI Figure S2f). Finally, Ans could significantly reduce the MG effects on the endothelium *in vitro* (the endothelial barrier [Fig. 4] and tight junctions [Fig. 3a–c]), and this phenomenon corresponded to the formation of fewer MG adducts in mouse tissue (Fig. 5), with less capillary leakage (the signature symptom of septic shock; Fig. 6c) and with a higher probability of survival in mice with experimental sepsis (Fig. 6d). Fig. 7 provides a graphical abstract of the main results.

## Discussion

Our results suggest that MG has a detrimental effect not only in chronic inflammatory diseases, as described previously, but also in the (hyper)acute environment of sepsis.^4^^,^^43^ We hypothesised that high MG plasma concentrations in early sepsis may impair endothelial integrity, as early mortality in sepsis is driven by septic shock^44^ characterised by ‘profound circulatory, cellular, and metabolic abnormalities’,^1^ including severe capillary leakage.^45^, ^46^, ^47^ It has been shown that activated macrophages are potentially involved in this excessive accumulation of MG in sepsis.^48^ We demonstrated that MG causes severe endothelial damage, enabling molecules with a molecular weight equal to that of albumin to escape from the capillary system into the interstitium. Beyond the frequently described direct toxic effects of MG on neighbouring molecules, we have shown that MG-derived AGEs impair capillary tightness via a RAGE/mitogen-activated protein kinase-mediated pathway and thus lead to the development of septic shock. These findings are consistent with our previous clinical data associating the concentration of the soluble RAGE with disease severity in patients with septic shock.^49^^,^^50^ It is also known that RAGE knockout in the CLP model increases the survival probability of mice.^51^ However, prior to the present study, it had not yet been shown that these observations are causally linked to MG stress, endothelial integrity, and the development of septic shock.

Oxidative stress is another major factor that contributes to endothelial dysfunction in inflammatory settings by activating the NLRP-3 inflammasome.^52^ MG, a highly reactive carbonyl species, exerts comparable effects on its surroundings.^53^ However, we found that MG-induced effects exceeded those of reactive oxygen species on the endothelial barrier since the potent antioxidant N-acetyl-cysteine was inferior to Ans at preventing an MG-induced loss of TER. Correspondingly, mitogen-activated protein kinases (p38 and c-Jun) were recently shown to decrease the expression of tight junction proteins via activation of the NLRP-3 inflammasome.^54^^,^^55^ By identifying the MG-AGE/RAGE axis, whose activation is associated with a loss of tight junction proteins and TER, we provide an important contributor to tight junction loosening in sepsis. Interestingly, this mechanism seems to be independent of NF-κB, a well-known driver of inflammation in sepsis.^56^ At the same time, this non-activation of NF-κB makes it highly unlikely that other pattern recognition receptors which are relevant in sepsis, such as the Toll-like receptor 4 (TLR4), are involved in the mechanism described here, although it is known that there is a lot of cross-talk in the innate immune response (in particular between RAGE and TLR4 and their downstream signalling pathways).^57^^,^^58^ The described MG effect in endothelial cells appears to be predominantly mediated by RAGE. Otherwise, it could hardly be explained why selective blockade of RAGE by FPS-ZM1 significantly reduced the MG effect on the TER as well as activation of signalling steps downstream of RAGE, such as MAP kinases (c-Jun). As MAP kinases are also activated by other pattern recognition receptors (PRRs), including the sepsis-relevant TLR4,^57^^,^^59^ one would expect them to be activated even after RAGE blockade if MG-AGEs were able to act via several of these receptors. Moreover, if MG or MG-AGE were able to activate RAGE as well as TLR4, one would expect that NF-κB, an important downstream element of the TLR4 pathway, would be activated in endothelial cells after MG stimulation. By contrast, we found that MG-mediated stimulation of endothelial cells was not associated with an activation of NF-κB, and RAGE blockade did not lead to MAP kinase activation and preserved the TER under MG stimulation. Consistently, another research group recently showed that, although TLR4 activation in rheumatoid arthritis leads to activation of endothelial cells, this phenomenon is not associated with endothelial dysfunction.^60^ Of course, in real-life (*in vivo*) sepsis, all of these pathways are activated simultaneously via different mechanisms, and they mutually reinforce each other. For example, activation of TLR4 can contribute to the increased formation of MG. Moreover, the interleukins formed as a result of MG-induced RAGE activation in turn activate immune cells, which then set off additional inflammatory cascades. MG, however, seems to have a particular influence on endothelial function via the signalling pathway described in this work. The link between MG-AGE and endothelial dysfunction has already been described for chronic diseases, because chronically elevated reactive carbonyl species–AGE concentrations impaired the blood-brain and blood-retinal barriers by disrupting tight junctions.^5^^,^^6^ However, a chronically increased but less severe MG load, such as in diabetes mellitus or uraemia, does not result in a massive capillary leak syndrome with subsequent shock induction.^61^ Accordingly, these MG effects appear to be dose-dependent and limited to the per acute setting of sepsis, in which a variety of inflammatory metabolic pathways are simultaneously upregulated and coactivated. MG, therefore, represents only one component in a network of metabolic pathways leading to the development of septic shock. However, MG is crucial, as confirmed by the MG-limiting sepsis experiments using Ans. In diabetes mellitus, aminoguanidine,^62^ Cns,^37^^,^^63^ and Ans^36^ antagonise MG-induced harmful effects *in vitro* and *in vivo*. Unfortunately, aminoguanidine increases mortality in experimental sepsis by suppressing the nitric oxide (NO) pathway.^64^ In the present study, the effects of Cns on endothelial stability in sepsis did not last as long and were not as pronounced effects as the effects of Ans. We have examined this superiority of Ans over Cns in terms of its protective properties against MG in another study and have concluded that these changes in chemical behaviour are most likely influenced by the methylation status of specific sites.^65^ In contrast, Ans reduced both MG- and LPS-induced impairments of endothelial integrity *in vitro* and *in vivo*. Moreover, Ans significantly reduced mortality in a murine model of experimental sepsis. Ans also exerts antioxidative effects, making it even more interesting for sepsis therapy since oxidative stress is another hallmark of early sepsis, with reactive oxygen species, reactive nitrogen species, and reactive carbonyl species being the major players.^26^^,^^66^^,^^67^

Our work has several strengths and limitations. The main strength is that we successfully combined patient data with *in vivo* and *in vitro* models, employing a translational approach. The clinical data used in the secondary analyses originated from observational studies that have been published in respected journals and, therefore, have already undergone a rigorous peer review process.^7^^,^^9^ However, both clinical studies used for secondary analyses within this work were exploratory and had a limited number of cases.^7^^,^^9^ Therefore, we carefully checked for possible confounding factors. For example, there was a median age difference of 3 years between the ‘lower half’ and the ‘upper half’. Although this was not statistically significant in the age distribution, we wanted to rule out the possibility that this was a potential confounding factor. We did not find any correlation between age and MG plasma concentrations, the survival time, or the degree of organ dysfunction reflected by the Sequential Organ Failure Assessment (SOFA) score (SI Table S1; SI Figure S1a–c).^7^ Likewise, there was no correlation between age and MG plasma concentrations in the sample as measured in the other secondary evaluation (SI Figure S1d).^9^ Moreover, we would like to point out that both observational studies used for secondary analyses in this work included patients with a high disease burden (reflected by high SOFA scores). Therefore, it is particularly interesting that in this high-risk group with high disease severity, it seems possible to predict a poor prognosis even at the onset of sepsis by using MG. Together with the causal relationship between MG and shock development shown in this work, this makes sense, because MG could contribute to the development of more severe forms of septic shock and thus increase mortality. Another strength of this work is that the observations in the cell culture models could be reproduced very well in the mouse models. For example, the experiments with EB in the mouse model showed very clearly that Ans could reduce capillary leakage in sepsis, just as it stabilises TER in the cell culture and reduces paracellular protein transport after MG stimulation. This translational approach helps to compensate for the inherent weaknesses of all sepsis research. Sepsis is a systemic, multifactorial disease that affects all organ systems and their complex interactions. At the onset of sepsis, a large number of PRRs are activated as part of the innate immune response, which in turn activates a large number of immune, endothelial, and epithelial cells, among other things. Subsequently, a multitude of different cytokines are released, often referred to in the literature as a cytokine storm. These activated pathways potentially interact with each other. Hence, it is extremely difficult to investigate individual pathways *in vivo*, and at the same time, it will always be imperfect to investigate only a single pathway *in vitro*. We have tried to show here, through some *in vivo* ‘proof-of-concept’ experiments, that the pathway identified *in vitro* has clinical relevance despite all the interactions of the various pathways. It would have been beyond the scope of this work to investigate the potential effect of Ans on each of the other metabolic pathways and the entire network. To test the partial relevance of each receptor that could potentially be activated in the network in mouse models would have resulted in the generation of a large number of necessary knock-out strains without any significant gain in knowledge. Instead, we focussed on the identification of MG as a biomarker associated in patients not only with the characteristic symptoms of septic shock but also with a particularly poor prognosis. We were able to identify a mechanism by which this biomarker causes endothelial damage, which in turn causally contributes to the development of shock symptoms. Then, we tested Ans, a drug that has already been shown to prevent and partially reverse MG-related damage in chronic diseases, in the context of sepsis. This had a clinical effect in the animal model (reduction of capillary leak and mortality). Meanwhile, the safety of histidine (HIS)-containing supplements such as Ans appear to be high in many conditions, such as neurodegenerative and age-related disorders, metabolic syndromes, or inflammatory bowel diseases.^68^, ^69^, ^70^, ^71^ Ans therefore represents a promising therapeutic approach for patients with sepsis, septic shock, or severe infections at high risk of progressing to sepsis.

## Contributors

TS conceived the study, performed data acquisition and data analysis, and wrote the manuscript. NG performed data acquisition and data analysis and was involved in writing the manuscript. VP and CPS were involved in the study's conception, hypothesis delineation, and design as well as in drafting the manuscript. AM, MB, and UK performed data acquisition and were involved in critically revising the manuscript. MF participated in the study design and revised the statistical analyses. PPN, FU, and MAW revised the study's conception and the manuscript critically. TS, NG, VP, MAW, CPS, and TB have accessed and verified the underlying data. TB conceived the study, participated in its design, coordinated the work group, and was involved in the writing process. All authors read and approved the final manuscript.

## Data sharing statement

The datasets generated and/or analysed during the current study are available from the corresponding author, Prof. Dr. Thorsten Brenner (thorsten.brenner@uk-essen.de) on reasonable request.

## Declaration of interests

The authors declare that they have no competing interests and that they have full control of all primary data.

## Appendix A Supplementary data

Supplementary Figures S1–S7, Methods M1, M2 and Tables S1–S5
