# Supplementary material for: Anserine reduces mortality in experimental sepsis by preventing methylglyoxal-induced capillary leakage
Source: eBioMedicine. 2025 Mar 18;114:105644. doi: 10.1016/j.ebiom.2025.105644 (PMC11995882; doi:10.1016/j.ebiom.2025.105644)
Supplement: Supplementary Figures S1–S7, Methods M1, M2 and Tables S1–S5 [file mmc1.pdf]

Supplementary Information
Schmoch Th. et al., Anserine reduces mortality in experimental sepsis by preventing methylglyoxal induced capillary leakage

| | | |
|---|---|---|
| **Supplemental Figure S1:** | Supplementary data on MG-induced carbonyl stress in septic patients and on TER under different inflammatory stimuli *in vitro*. | **Page 2** |
| **Supplemental Figure S2:** | Additional toxicity and functionality tests. | **Page 3** |
| **Supplemental Figure S3:** | Inflammatory cytokine profile from MG- and Ans stimulated HUVEC. | **Page 4** |
| **Supplemental Figure S4:** | Effects of supraphysiological MG and Ans cotreatment and cotreatments with Cns, AG and NAC on the TER of a HUVEC monolayer. | **Page 5** |
| **Supplemental Figure S5:** | Effect of common MG-scavengers on endothelial barrier proteins. | **Page 6** |
| **Supplemental Figure S6:** | Effects of Ans treatment on EB extravasation in septic mice. | **Page 7** |
| **Supplemental Figure 7:** | *In vivo* Ans tissue concentrations of septic mice. | **Page 8** |
| **Supplemental Methods M1:** | Mouse models | **Page 9** |
| **Supplemental Methods M2:** | Sepsis Definitions | **Page 10** |
| **Supplemental Table S1:** | List of materials | **Page 11** |
| **Supplemental Table S2:** | Overview of the design of the individual experiments and the numbers of animals used. | **Page 13** |
| **Supplemental Table S3:** | Baseline characteristics of patients included from Brenner T. et al. | **Page 14** |
| **Supplemental Table S4:** | Baseline characteristics of patients included from Nusshag C. et al. | |
| | a) Baseline characteristics of a random sample of 20 patients stratified by their plasmatic MG concentrations at intensive care unit (ICU) admission. | **Page 15** |
| | b) Baseline characteristics of the abovementioned random sample of 20 patients as compared to the rest of the cohort (n = 80). | **Page 16** |
| **Supplemental Table S5:** | Supportive statistics from MG, LPS and TNF titration | **Page 17** |
| **References for Supplementary Information** | | **Page 18** |

Supplementary Information

Schmoch Th. et al., Anserine reduces mortality in experimental sepsis by preventing methylglyoxal induced capillary leakage

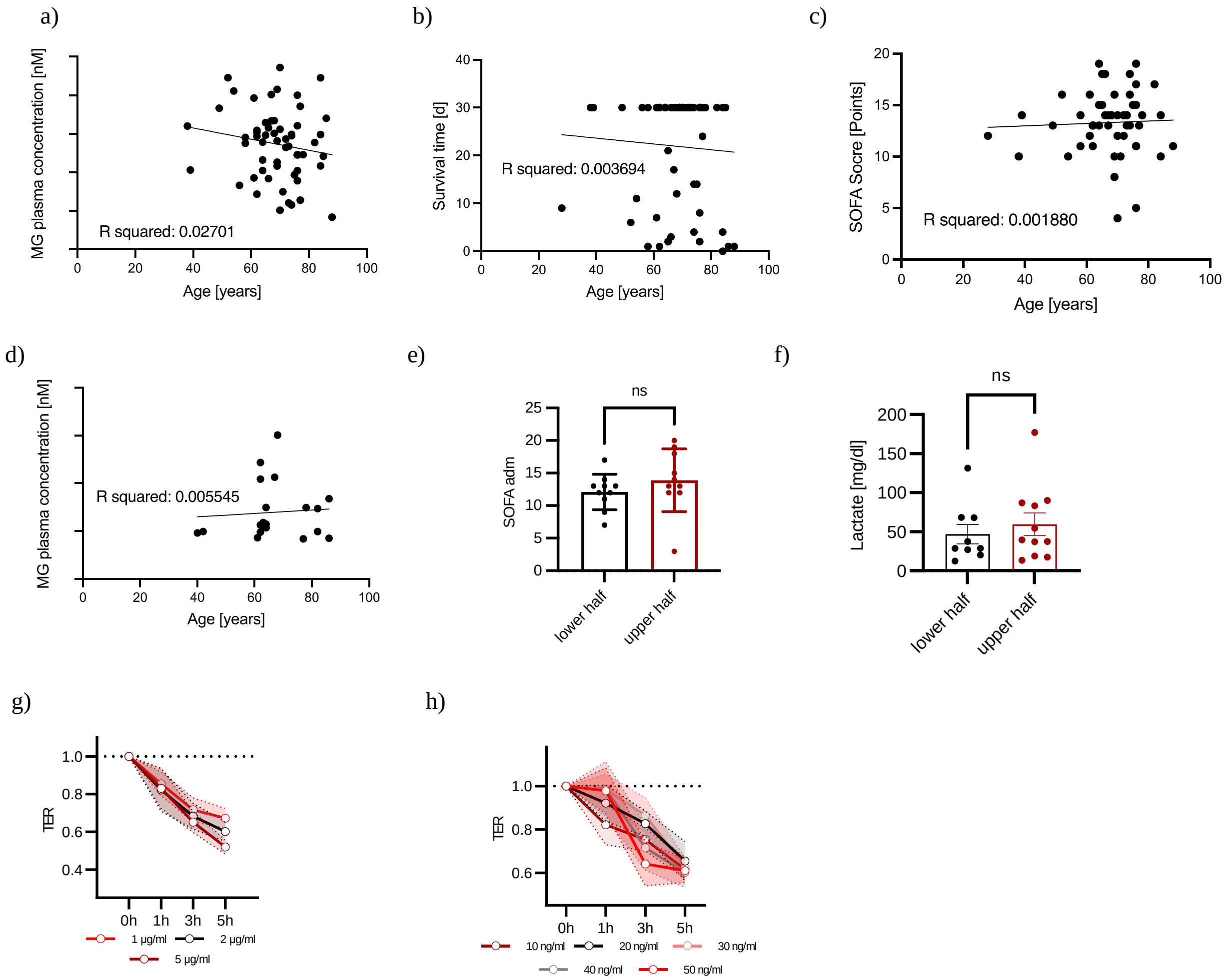


**Supplemental Figure S1: Supplementary data on MG-induced carbonyl stress in septic patients and on TER under different inflammatory stimuli *in vitro*.**

a) Correlation of age and MG plasma concentrations at sepsis onset in patients from Brenner Th et al.[1]

b) Correlation of age and survival time in patients from Brenner Th et al. [1]

c) Correlation of age and SOFA Socre at sepsis onset in patients from Brenner Th et al.[1]

d) Correlation of age and MG plasma concentrations at sepsis onset in patients from Nusshag Ch. et al. [2]

e) SOFA scores and

f) plasmatic lactate [mg/dl] concentrations at ICU-admission (= sepsis onset) in 50% of septic patients with highest MG plasma concentrations (= upper half, n = 10) vs. 50% of septic patients with lower MG plasma concentrations (= lower half, n = 10). Mann-Whitney U-test. Data represent mean and SEM. Secondary analysis from Nusshag C. et al.[2.]

g) Time course of 5 h TER experiment of HUVEC monolayers under single LPS-treatment (n = 6) and

h) time course of 5 h TER experiment of HUVEC monolayers under single TNF-treatment (n = 6). Data represent mean and SEM.

Concerning symbols: ns = not significant

Abbreviations: HUVEC; human umbilical vein endothelial cell; ICU, intensive care unit; LPS, lipopolysaccharide; MG, methylglyoxal; SEM, standard error mean; SOFA, Sequential Sepsis-related Organ Failure Assessment; TER, transendothelial electrical resistance.

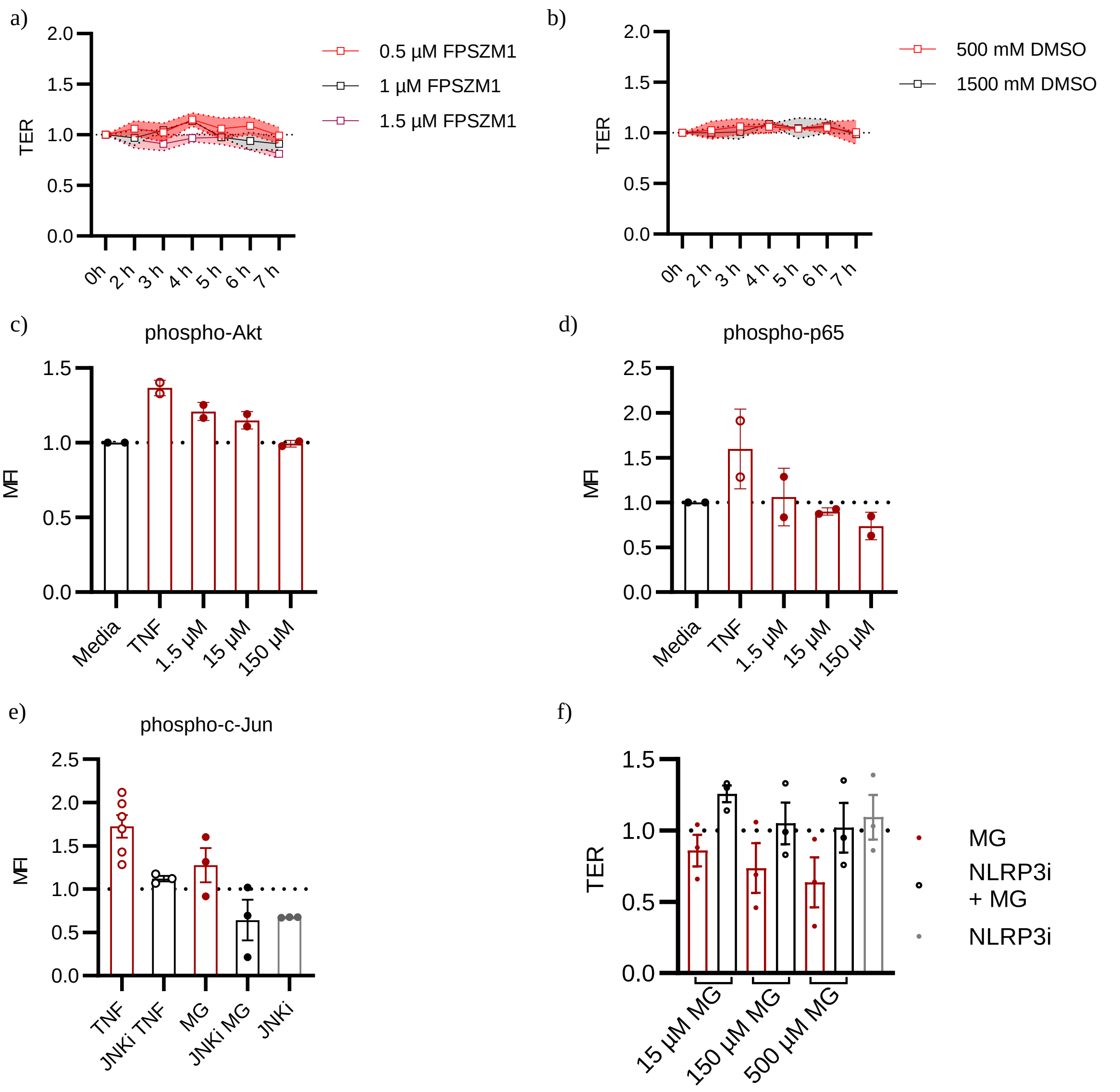


**Supplemental Figure S2: Additional toxicity and functionality tests.**

a) Time course of 5 h trans endothelial electrical resistance (TER) experiments in HUVEC following a 2 h pretreatment with FPSZM1 in 100 DMSO only. Data represent mean and SEM.

b) Complementary time course to A) of 5 h TER experiments in HUVEC following a 2 h pretreatment with DMSO solvent control only. Data represent mean and SEM.

c) Phospho-Akt Fluorescence-activated cell sorting (FACS) staining in HUVEC after single treatments with MG or TNF (50 ng/ml). Data represent mean and SEM.

d) Phospho-p65 (NF-κB subunit) FACS staining in HUVEC after single treatments with MG or TNF (50 ng/ml). Data represent mean and SEM.

e) Phospho-c-Jun FACS staining in HUVEC after singe treatments with 150 µM MG or TNF (50 ng/ml) for 30 min and after 20 h JNKi SP600125 pretreatment and/or a single dose of MG or TNF for 30 min. Data represent mean and SEM.

f) Time course of 5 h TER experiments in HUVEC following a 3 h pretreatment with NLRP3i MCC950 (50µM) and single treatments with MG for 2 h. Data represent mean and SEM.

Abbreviations: Akt, protein kinase B; c-Jun, c-Jun N-terminal kinase; DMSO, dimethyl sulfoxide; Fluorescence-activated cell sorting (FACS), JNKi, c-Jun N-terminal kinase inhibitor; MG, methylglyoxal; NLRP3i, nucleotide-binding oligomerization domain (NOD), leucine-rich repeat (LRR)-containing protein (NLR) family member (NLRP3) inhibitor; SEM, standard error mean; TER, transendothelial electrical resistance; TNF, tumor necrosis factor.

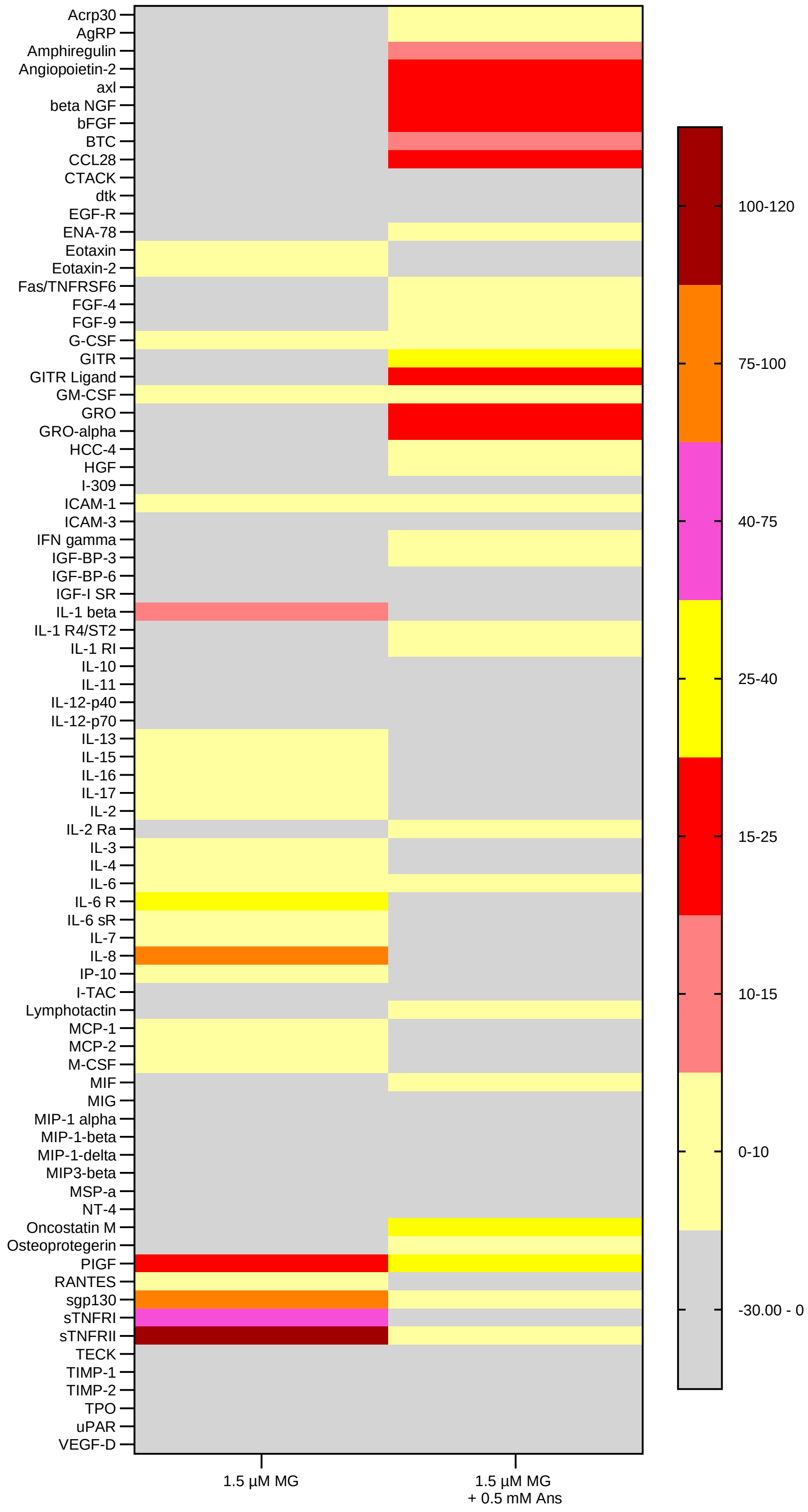


**Supplemental Figure S3: Inflammatory cytokine profile from MG- and Ans stimulated HUVEC.**

Cytokine array measuring cytokine protein concentrations of inflammation-, apoptosis-, and angiogenic-related cytokines, excreted from HUVEC monolayer after incubation with MG for 5 h in comparison to MG-free media control. MG was administered every 2 h, Ans was administered once together with the first MG treatment. Heat map represents dot intensity difference of treatment and control values of batched supernatants from n = 4 experiments. Scale on the right side: Fold change.

Abbreviations: Ans, anserine; HUVEC, human umbilical vein endothelial cell; MG, methylglyoxal.

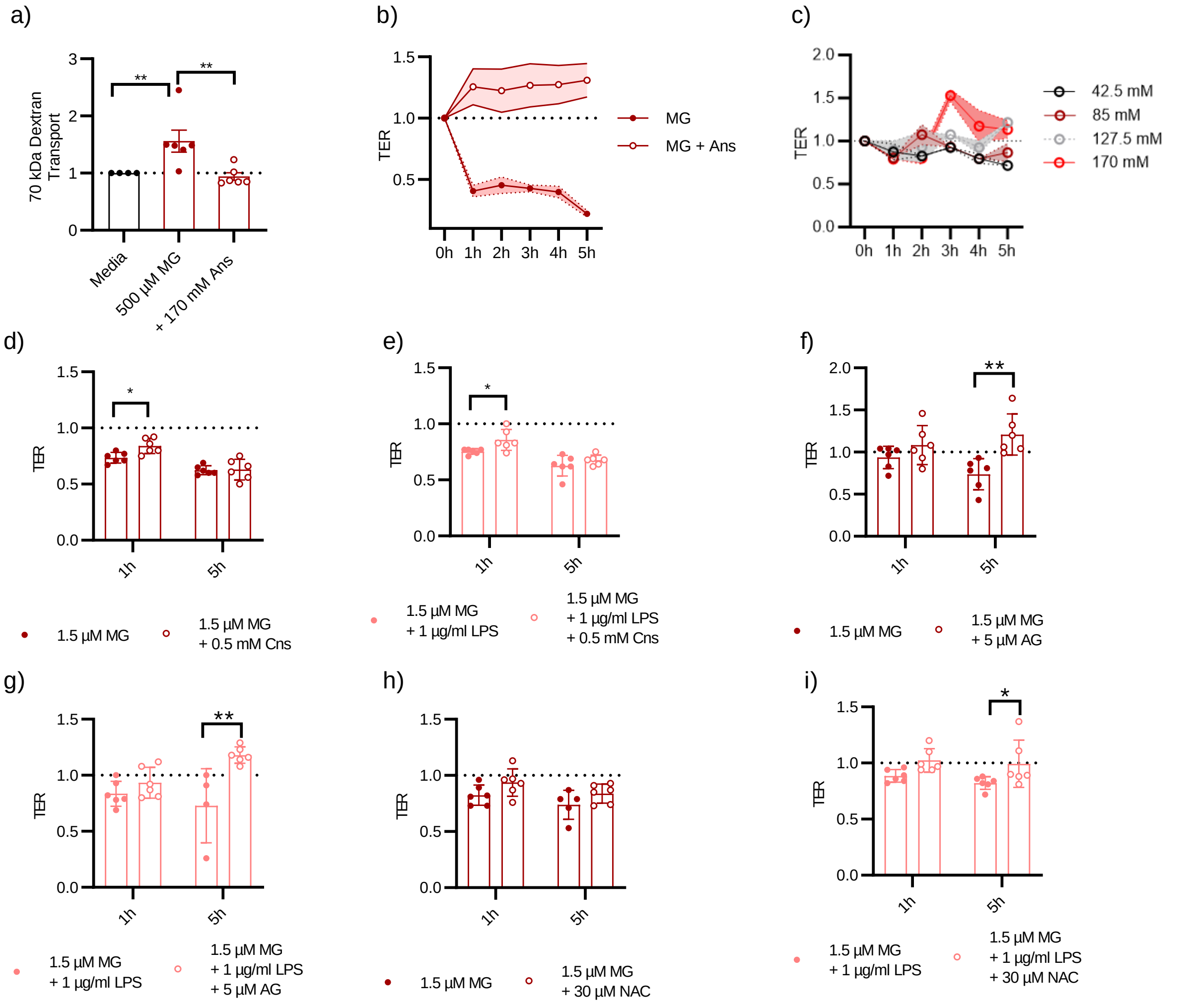


**Supplemental Figure S4: Effects of supraphysiological MG and Ans cotreatment and cotreatments with Cns, AG and NAC on the TER of a HUVEC monolayer.**

a) Paracellular leak of 70 kDa dextran through a primary HUVEC monolayer following repetitive administrations of supraphysiological MG only or with Ans cotreatments (control vs. MG: $p$ = 0.009; MG vs. MG + Ans: $p$ = 0.004) at 5 h. Data represent mean and SEM. Mann-Whitney U-test
b) Time course of TER of a HUVEC monolayer under repetitive supraphysiological MG (500 µM) and Ans (170 mM) cotreatments over 5 h. Data represent mean and SEM.
c) Ans dose escalation. Data represent mean and SEM.
d) Effect of MG and cotreatment with Cns on the TER of a HUVEC polarized monolayer (1 h, MG vs MG + Cns: $p$ = 0.02).
e) Effect of MG with concomitant LPS and Cns stimulation (1 h, MG +LPS vs. MG + LPS + Cns: $p$ = 0.03).
f) Effect of MG and cotreatment with AG on the TER of a HUVEC polarized monolayer (5 h, MG vs. MG + AG: $p$ = 0.002).
g) Effect of MG with concomitant LPS and AG stimulation (5 h, MG + LPS vs. MG + LPS + AG: $p$ = 0.009).
h) Effect of MG and cotreatment with NAC on the TER of a HUVEC polarized monolayer.
i) Effect of MG with concomitant LPS and NAC stimulation (5 h, MG +LPS vs. MG + LPS + NAC: $p$ = 0.04).

d-i) MG was administered every 2 h, LPS, Cns, AG and NAC were administered once together with the first MG treatment. All data represent mean and SEM. Mann-Whitney U-test.

Concerning symbols: * $p \leq 0.05$; ** $p < 0.01$.

Abbreviations: AG, aminoguanidine; Ans, anserine; Cns, carnosine; HUVEC, human umbilical vein endothelial cell; LPS, lipopolysaccharide; MG, methylglyoxal; NAC, N-acetylcysteine, SEM, standard error mean; TER, transendothelial electrical resistance.

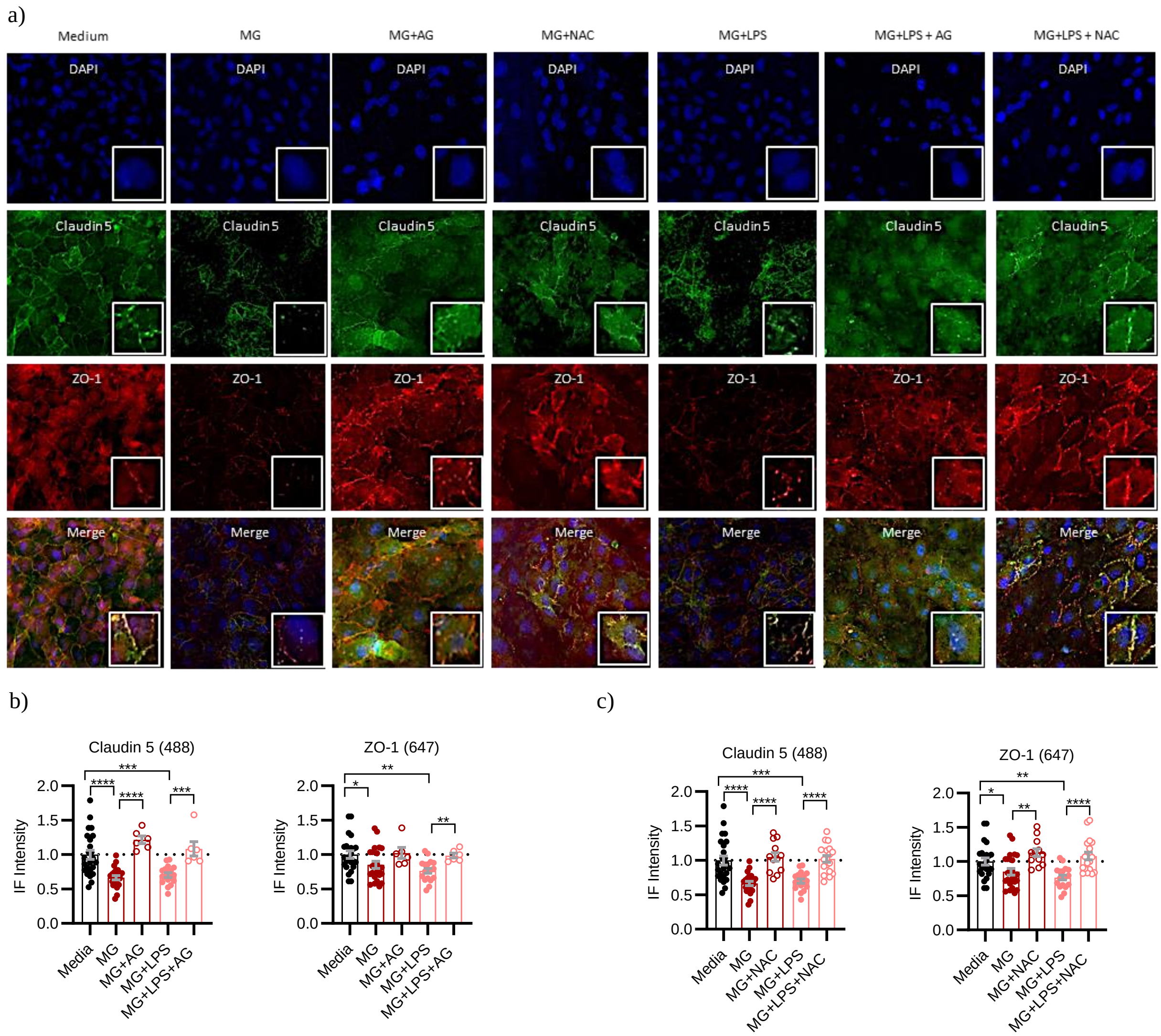


**Supplemental Figure S5: Effect of common MG-scavengers on endothelial barrier proteins.**

a) Representative immune fluorescence claudin 5 and ZO-1 staining in HUVEC treated (from left to right) with media, 1.5 µM MG, 1.5 µM MG + 5 µM AG, 1.5 µM MG + 30 µM NAC, 1.5 µM MG + 1 µg/ml LPS, 1.5 µM MG + 1 µg/ml LPS + 5 µM AG and 1.5 µM MG + 1 µg/ml LPS + 30 µM NAC for 5 h.

b) Bar graph of relative IF intensity of HUVEC stained for claudin 5 and ZO-1. HUVEC were treated with media, 1.5 µM MG, 1.5 µM MG + 5 µM AG, 1.5 µM MG + 1 µg/ml LPS or 1.5 µM MG + 1 µg/ml LPS + 5 µM AG for 5 h. Claudin 5: control vs. MG: $p \leq 0.0001$; control vs. MG + LPS: $p = 0.0003$; MG vs. MG + AG: $p \leq 0.0001$; MG + LPS vs. MG + LPS + AG: $p = 0.0002$. ZO-1: control vs. MG: $p = 0.0298$; control vs. MG + LPS: $p = 0.0011$; MG + LPS vs. MG + LPS + AG: $p = 0.0013$. Each bar represents the mean IF intensity relative to media control.

c) Bar graph of relative IF intensity of HUVEC stained for claudin 5 and ZO-1. HUVEC were treated with media, 1.5 µM MG, 1.5 µM MG + 30 µM NAC, 1.5 µM MG + 1 µg/ml LPS or 1.5 µM MG + 1 µg/ml LPS + 30 µM NAC for 5 h. Claudin 5: control vs. MG: $p \leq 0.0001$; control vs. MG + LPS: $p = 0.0003$; MG vs. MG + NAC: $p \leq 0.0001$; MG + LPS vs. MG + LPS + NAC: $p \leq 0.0001$. ZO-1: control vs. MG: $p = 0.0298$; control vs. MG + LPS: $p = 0.0011$; MG vs. MG + NAC: $p = 0.013$; MG + LPS vs. MG + LPS + NAC: $p \leq 0.0001$. Each bar represents the mean IF intensity relative to media control.

a-c) MG was administered every 2 h, LPS, AG and NAC were administered once together with the first MG treatment. All data represent mean and SEM. Mann-Whitney U-test.

Note: in subfigure S5a, columns 1, 2 and 5 are identical to those shown in figure 3.

<u>Concerning symbols</u>: * $p \leq 0.05$; ** $p \leq 0.01$; *** $p \leq 0.001$; **** $p \leq 0.0001$
Abbreviations: AG, aminoguanidine; HUVEC, human umbilical vein endothelial cell; IF, immune fluorescence; LPS, lipopolysaccharide; MG, methylglyoxal; NAC, N-acetylcysteine, SEM, standard error mean.

a)

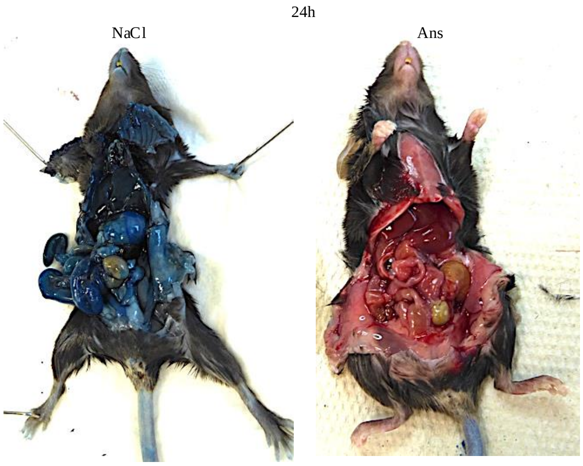


b)

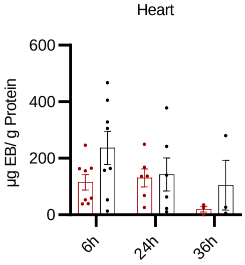


**Supplemental Figure S6: Effects of Ans treatment on EB extravasation in septic mice.**

a) Representative images of septic mice following CLP injected with EB at 24 h, prior to organ harvesting. Mice received an i.p. Ans or vehicle (0.9% NaCl) treatment.

b) Summary of EB extravasation in the heart stroma of septic mice. Mice were sacrificed at 6 h, 24 h and 36 h. Data represent mean and SEM. Mann-Whitney U-test.

Abbreviations: Ans, anserine; CLP, cecal ligation and puncture; EB, Evans blue; i.p., intraperitoneal.

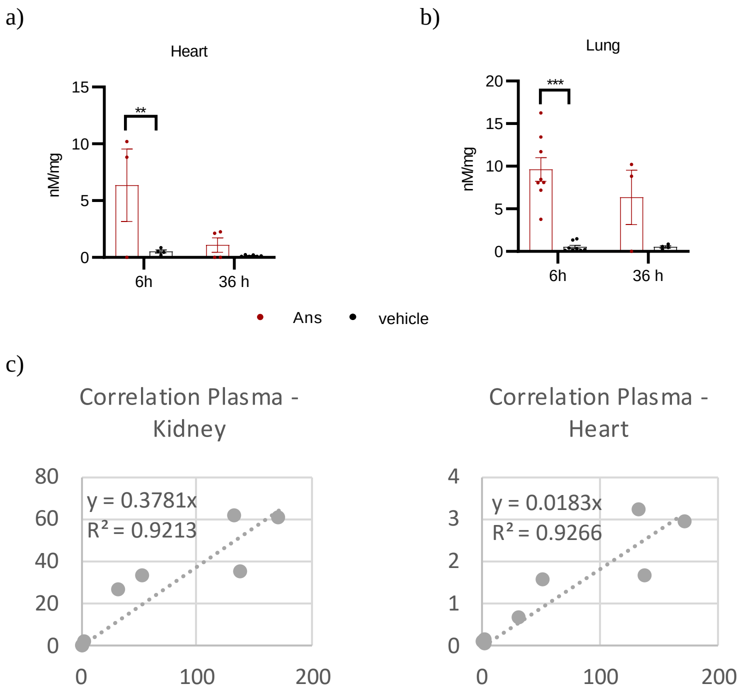


**Supplemental Figure 7: *In vivo* Ans tissue concentrations of septic mice.**

a) Ans concentrations in heart tissue samples [nM/mg] of septic C57/BL6 mice at 6 h (n = 8, $p$ = 0.001) and 36 h (n = 4) following CLP after i.p. Ans (1,500 mg/kg BW) or vehicle treatment (NaCl 0.9%). Measurements of Ans were performed using HPLC. Data represent mean and SEM. Mann-Whitney U-test.

b) Ans concentrations in lung tissue samples [nM/mg] of septic C57/BL6 mice at 6 h (n = 8, $p$ = 0.0003) and 36 h (n = 6) following CLP after i.p. Ans (1,500 mg/kg BW) or vehicle treatment (NaCl 0.9%). Measurements of Ans were performed using HPLC. Data represent mean and SEM. Mann-Whitney U-test.

c) Correlation analyses of Ans tissue/plasma concentrations of septic C57/BL6 mice (n = 7) following CLP after i.p. Ans (1,500 mg/kg BW) or vehicle treatment (NaCl 0.9%) at 24 h.

Concerning symbols: ** $p \leq 0.01$; *** $p \leq 0.001$
Abbreviations: Ans, anserine; CLP, cecal ligation and puncture; HPLC, high performance liquid chromatography; SEM, standard error mean.

## Supplemental Methods M1: Mouse models

**Study design and sample size**

The study design and the exact number of mice allocated to each group, and the total number in each experiment as well as the total number of animals used can be found in Table S2.

**Sample size planning in survival experiments**: Placebo-treated C57BL/6 mice had a mortality rate of about 50% within 7 days following CLP: $p_1$=0.5. It was expected that the mortality rates in the individual study groups differ by 40% from the respective control group (Dp=0.4) by the end of the 7-day study period. This results in a sample size of n=20 plus 2 (10%) dropouts in each case. The sample size calculation was based on the planned evaluation strategy using the log-rank test. It was carried out by the Institute of Medical Biometry (Heidelberg University, Heidelberg, Germany) using the planning software PROC POWER (SAS, Heidelberg, Germany).

**Sample size planning for the determination of carbonyl stress in experimental sepsis:** It was expected that there would be an effect size (e.g. difference in MG plasma concentration) of ≥1.4 between mice after CLP or LPS injection and the baseline, as well as in sham-operated animals. Parameters used: effect size: 1.4; standard deviation at baseline 0.8; standard deviation in the CLP group 0.8; nominal power 0.8; nominal alpha error 0.05. These calculations now yield a case number of n = 9 per study group. Furthermore, 10% (1) dropouts were expected in the procedures (CLP, sham surgery, injection of LPS). No dropouts were calculated for the "baseline" time point (= no surgical procedure).

The Institute of Medical Biometry (Heidelberg University, Heidelberg, Germany) used a t-test-based sample size calculation (for 2 independent samples) as the basis for calculation (although the analysis was later to be carried out using the Wilcoxon test). Since each group was only to be compared with the CLP group, a sample size calculation with the two-tailed t-test was considered sufficient. The comparison of all groups against each other is not possible with the selected procedure/group size, but is also not necessary to answer the research question.



**Inclusion and exclusion criteria**

**Scoring:** The animals were assessed every eight hours according to a predefined scoring system that had been approved in advance by the relevant authorities. If the score exceeded nine points, the animal was removed from the experiment and euthanised. The score assessed the appearance of the fur and posture, breathing rate, weight loss, escape behaviour and the integrity of the abdominal suture.

**Randomisation and blinding**

The mice were randomised to a treatment arm immediately after CLP. Randomisation was performed by a person other than the person who had performed CLP and who was blinded to the course of CLP. The RAGE-/-GLO+/+ status was blinded to the person who performed the CLP. The scoring of the mice (described above) was carried out in a blinded manner with respect to the treatment arm or RAGE-/-GLO+/+ status.

**Outcome measures**

Table S2 lists the outcome measures of the individual experiments.

**Experimental animals**

Female C57BL/6J mice (10–12 weeks of age, 18–22 g/mouse) were purchased from Charles River. Female C57BL/6N RAGE−/− GLO+/+ mice and the corresponding wildtype control animals from the same breed (10–12 weeks of age, 18–22 g/mouse) were provided by the laboratory of the Department of Medicine at the Heidelberg University (Heidelberg, Germany) and characterized by Bartling et al.[3]

**General information**

All mice were maintained in a pathogen-free environment and housed in clear shoebox cages in groups of three animals per cage with constant temperature (20-22 °C) and humidity and 12 h:12 h light-dark cycle, within a specialized facility for interfaculty biomedical research at Heidelberg University. All animals always had access to water and were fed *ad libitum*. Following CLP or LPS injection, the animals received painkillers (opioids) every 8 hours.

## Supplemental Methods M2: Sepsis Definitions

In this article, the term “sepsis” refers to sepsis as defined by the “Third International Consensus Definitions for Sepsis and Septic Shock (Sepsis-3)”. This defines sepsis as “life-threatening organ dysfunction caused by a dysregulated host response to infection” [4.] “Septic shock is a subset of sepsis in which underlying circulatory and cellular/metabolic abnormalities are profound enough to substantially increase mortality“.[4] “Patients with septic shock can be identified with a clinical construct of sepsis with persisting hypotension requiring vasopressors to maintain MAP ≥65 mm Hg and having a serum lactate level >2 mmol/L (18mg/dL) despite adequate volume resuscitation“.[4] „With these criteria, hospital mortality is in excess of 40%“.[3] By the expression “severe infection”, the authors refer to infections that could potentially lead to sepsis according to the above definition. In particular this includes infections that have caused a systemic inflammatory response syndrome (SIRS) but have not (yet) led to organ dysfunction. According to the Sepsis-2 definitions valid until 2016, such infections accompanied by SIRS were considered “sepsis” already.[5] This was changed with the Sepsis-3 definitions, since a systemic inflammatory response syndrome is a potentially useful host response to a generalized infection. However, sepsis is characterized by a dysregulated host response.[4] The term “course of disease” refers to the probability of an increase in organ failure (measured in humans using the Sequential (sepsis-related) Organ Failure Assessment (SOFA) score[4]), the probability of progression from sepsis to septic shock, and the mortality rate. In this article, the term“ disease severity” refers to the degree of organ dysfunction (measured using the SOFA score) +/- the presence of septic shock.

## Supplemental Table S1: List of materials

| Commercial Kits | Catalogue number | Company |
|---|---|---|
| Caspase-Glo® 1 Inflammasome Assay | G9951 | Promega<br>Madison, USA |
| Pierce lactatedehydrogenase (LDH) Cytotoxicity Assay Kit | 88953 | Thermo Fisher Scientific<br>Waltham, USA |
| Human Cytokine Array C3 and C7 | AAH-CYT-3-2<br>AAH-CYT-7-2 | RayBiotech Life<br>Peachtree Corners, USA |
| Endothelial Cell Growth Medium Kit | C-22010 | PromoCell - Heidelberg, DE |

**Antibodies and dyes**

| Target | Catalogue number | RRID | Reference to validation information | Company |
|---|---|---|---|---|
| Anti-ZO-1 ZO-1 Monoclonal Antibody (ZO1-1A12), Alexa Fluor™ 647 | MA3-39100-A647 | AB_3093163 | 6 | Thermo Fisher Scientific<br>Waltham, USA |
| PE Mouse anti-NF-κB p65 (pS529) | 558423 | AB_647222 | 7 | BD<br>Franklin Lakes, USA |
| Claudin 5 Monoclonal Antibody (4C3C2), Alexa Fluor™ 488, Invitrogen™ | ab131259 | AB_1115794<br>0 | 8 | Thermo Fisher Scientific<br>Waltham, USA |
| Phospho-c-Jun (Ser73) (D47G9) XP® Rabbit mAb (PE Conjugate) | #8752 | AB_2797656 | 9 | Cell Signaling Technology<br>Danvers, USA |
| Phospho-Akt (Ser473) (D9E) XP® Rabbit mAb (Alexa Fluor® 647 Conjugate) | #4075 | AB_916029 | 10 | Cell Signaling Technology<br>Danvers, USA |
| Phospho-p38 MAPK (Thr180/Tyr182) (28B10) Mouse mAb (Alexa Fluor® 488 Conjugate) | #4551 | AB_331302 | 11 | Cell Signaling Technology<br>Danvers, USA |
| Phospho-IRF-3 (Ser386) (E7J8G) XP® Rabbit mAb (Alexa Fluor® 647 Conjugate) | #96421 | AB_2800264 | 12 | Cell Signaling Technology<br>Danvers, USA |
| Anti-AGE Rabbit pAb | ab23722 | AB_447638 | 13 | abcam<br>Cambridge, UK |
| 4',6-diamidino-2-phenylindol (DAPI) | D9542 | n/a | n/a | Sigma- Aldrich<br>St. Louis, USA |

**Other**

| Reagent | Catalogue number | Company |
|---|---|---|
| 40 % Methylglyoxal | 67028 | Sigma-Aldrich,<br>St. Louis, USA |
| LPS ultrapure | tlrl-3pelps | InvivoGen<br>San Diego, USA |
| L-Anserine | J97346 | AK Scientific, Inc<br>Union City, USA |
| L-Carnosine | C9625 | Sigma-Aldrich,<br>St. Louis, USA |
| rhTNFa | #300-01A | PeproTech, Inc.<br>Rocky Hill, USA |
| N-Acetylcysteine | A7250 | Sigma-Aldrich,<br>St. Louis, USA |
| Aminoguanidine | 396494 | Sigma-Aldrich,<br>St. Louis, USA |
| Fluorescein isothiocyanate (FITC)-Dextran (4, 10, 70 kDa) | 00271, 31390, 90718 | Sigma-Aldrich<br>St. Louis, USA |
| Dextran (10, 70 kDa) | 46944, FD10S | Sigma-Aldrich,<br>St. Louis, USA |
| Trypan blue 0.4 % | 93595 | Sigma- Aldrich<br>St. Louis, USA |
| FPS ZM1 | 553030 | Sigma- Aldrich<br>St. Louis, USA |

**Supplemental Table S1: List of materials**

**Animals, drugs, and reagents for animal experiment**

| | | |
|---|---|---|
| C57/BL6 | 632C57BL/6J | Charles River |
| Evans blue | A16774-18 | Thermo Fisher Scientific<br>Waltham, USA |
| Micro-Osmotic Pump | Model 1007D | ALZET<br>Cupertino, USA |

**Devices**

| | |
|---|---|
| Tecan Infinite F200 Fluorescence Microplate Reader | Tecan Group AG<br>Männedorf, CH |
| Acquifer Imaging Machine | ACQUIFER Imaging<br>Heidelberg, DE |
| Tecan Spark® Multimode Microplate Reader | Tecan Group AG<br>Männedorf, CH |
| Fluorescence activated cell sorting (FACS) lyric | BD<br>Franklin Lakes, USA |
| Chemiluminescence imaging systems, Fusion FX7 | Vilber<br>Collégien, FR |

**Supplemental Table S2: Overview of the design of the individual experiments and the numbers of animals used.**

| Experimental setup | Test groups | Number of test animals | Target values |
|---|---|---|---|
| **Project part 1** (shown in Figure 1b) | | | |
| **Section 1** | | | |
| Blood sampling in C57BL/6J mice 36h following Sham-OP or CLP | Baseline | 7 | MG plasma concentration at baseline vs. 36h following CLP or sham surgery |
| | Sham-OP 36h | 8 | |
| | Mild CLP 36h | 8 | |
| **Section 2** | | | |
| Blood sampling in C57BL/6J mice 6h, 16h and 36h following LPS administration | LPS 6h | 8 | MG plasma concentration at baseline and at 6h, 16h and 36h following LPS injection |
| | LPS 16h | 8 | |
| | LPS 36h | 8 | |
| **Project part 2** (shown in Figure 1c) | | | |
| Survival experiment following CLP with subsequent treatment of C57BL/6J mice with placebo or MG | CLP + placebo (NaCl 0.9%) | 16 | Survival after 7d |
| | CLP + MG | 16 | |
| **Project part 3** | | | |
| **Section 1** (shown in Figure 3d): | | | |
| Blood sampling in C57BL/6 RAGE$^{-/-}$+GLO$^{+/+}$ vs C57BL/6N wildtype mice 6h, 16h and 36h following CLP | 6h<br>C57BL/6N RAGE$^{-/-}$+GLO$^{+/+}$<br>C57BL/6N wildtype | <br>7<br>7 | MG plasma concentration 6h, 16h and 36h follwing CLP |
| | 16h<br>C57BL/6N RAGE$^{-/-}$+GLO$^{+/+}$<br>C57BL/6N wildtype | <br>7<br>7 | |
| | 36h<br>C57BL/6 RAGE$^{-/-}$+GLO$^{+/+}$<br>C57BL/6N wildtype | <br>7<br>7 | |
| **Section 2** (shown in Figure 3e): | | | |
| Survival experiment | C57BL/6N RAGE$^{-/-}$+GLO$^{+/+}$ | 9 | Survival after 7d |
| | C57BL/6N wildtype | 15 | |
| **Project part 4** | | | |
| **Section 1** (shown in Figures 5 and 6 a + b): | | | |
| Anserine or placebo treatment and subsequent organ harvesting in C57BL/6 mice at 6h, 24h and 36h following CLP | 6h<br>C57BL/6J + placebo<br>C57BL/6J+ Anserin | <br>7<br>7 | Capillary leak and MG-AGE tissue concentration 6h, 24h and 36h following CLP |
| | 24h<br>C57BL/6J + placebo<br>C57BL/6J+ Anserin | <br>7<br>7 | |
| | 36h<br>C57BL/6J + placebo<br>C57BL/6J+ Anserin | <br>7<br>7 | |
| **Section 2** (shown in Figure 3e): | | | |
| Survival experiment | C57BL/6J + placebo | 22 | Survival after 7d |
| | C57BL/6J + Anserine | 20 | |
| **Total number** | | **229** | |

**Supplemental Table S3: Baseline characteristics of patients included in an observational prospective clinical study (n = 60) from Brenner T. et al.[5].**

| | **Lower half** | | **Upper half** | | **p** |
|---|---|---|---|---|---|
| Sample size [n (%)] | 30 | (50.0) | 30 | (50.0) | |
| Age [median (min - max)] (years) | 72.0 | (39.0 – 88.0) | 67.0 | (28.0 – 86.0) | 0.1 |
| Sex [n (%)] | | | | | |
| male | 25 | (83.0) | 21 | (70.0) | 0.2 |
| female | 5 | (17.0) | 9 | (30.0) | |
| BMI [median (IQR)] (kg/m²) | 25.6 | (22.1 – 28.6) | 26.4 | (23.2 – 28.4) | 0.8 |
| SOFA sore at admission [median (IQR)] (points) | 14.0 | (11.0 – 16.0) | 13.0 | (11.5 – 14.0) | 0.3 |
| SAPS II sore at admission [median (IQR)] (points) | 74.5 | (62.3 – 86.8) | 77.0 | (71.0 – 86.0) | 0.6 |
| APACHE II score at admission [median (IQR)] (points) | 33.0 | (29.0 – 41.0) | 32.0 | (26.3 – 36.8) | 0.5 |
| Plasmatic lactate at admission [median (IQR)] (mg/dl) | 12.0 | (9.0 – 38.0) | 19.0 | (16.3 – 33.5) | 0.4 |

No data on on race or ethnicity was collected. Abbreviations: APACHE, Acute Physiology and Chronic Health Evaluation; BMI, Body Mass Index; IQR, inter quartile range; SAPS, Simplified Acute Physiology Score; SOFA, Sequential Sepsis-related Organ Failure Assessment

**Supplemental Table S4: Baseline characteristics of patients included in an observational prospective clinical study (n = 100) from Nusshag C. et al..[8]**

a) Baseline characteristics of a random sample of 20 patients stratified by their plasmatic MG concentrations at intensive care unit (ICU) admission.

| | Lower half | | Upper half | | p |
|---|---|---|---|---|---|
| Sample size [n (%)] | 10 | (10.0) | 10 | (10.0) | |
| Age [median (min - max)] (years) | 62.5 | (40.0 – 86.0) | 65.5 | (62.0 – 86.0) | 0.2 |
| Sex [n (%)] | | | | | |
| male | 7 | (70.0) | 7 | (70.0) | >0.9 |
| female | 3 | (30.0) | 3 | (30.0) | |
| BMI [median (IQR)] (kg/m$^2$) | 27.7 | (24.2 – 29.3) | 27.7 | (24.2 – 29.3) | 0.3 |
| Primary source of infection [n (%)] | | | | | |
| Abdomen | 8 | (80.0) | 6 | (60.0) | 0.8 |
| Lung | 1 | (10.0) | 2 | (20.0) | |
| Urinary Tract | 1 | (10.0) | 2 | (20.0) | |
| Others | 0 | (0.0) | 1 | (10.0) | |
| Combination | 1 | (10.0) | 1 | (10.0) | |
| Coexisting conditions [n (%)] | | | | | |
| Chronic kidney disease | 5 | (50.0) | 2 | (20.0) | 0.4 |
| Hypertension | 7 | (70.0) | 8 | (80.0) | |
| Diabetes mellitus | 1 | (10.0) | 4 | (40.0) | |
| Coronary heart disease | 3 | (30.0) | 2 | (20.0) | |
| Cancer | 4 | (40.0) | 7 | (40.0) | |
| | | | | | |
| SOFA sore at admission [median (IQR)] (points) | 12.5 | (11.3 – 13.0) | 13.5 | (9.5 – 14.5) | 0.1 |
| Plasmatic lactate at admission [median (IQR)] (mg/dl) | 28.6 | (22.0 – 60.5) | 47.0 | (17.7 – 75.7) | 0.2 |

No data on on race or ethnicity was collected. Abbreviations: BMI, Body Mass Index; IQR, inter quartile range; SOFA, Sequential Sepsis-related Organ Failure Assessment

**Supplemental Table S4: Baseline characteristics of patients included in an observational prospective clinical study (n = 100) from Nusshag C. et al..[8]**

b) Baseline characteristics of the abovementioned random sample of 20 patients as compared to the rest of the cohort (n = 80).

| | Measured samples | | Rest of the cohort | | p |
|---|---|---|---|---|---|
| Sample size [n (%)] | 20 | (20.0) | 80 | (80.0) | |
| Age [median (min - max)] (years) | 64 | (40 – 86) | 70.5 | (22 – 81) | 0.7 |
| Sex [n (%)] | | | | | |
| male | 14 | (70.0) | 17 | (85.0) | |
| female | 6 | (30.0) | 3 | (15.0) | |
| BMI [median (IQR)] (kg/m²) | 27.7 | (24.2 – 29.3) | 26.2 | (24.1 – 29.0) | 0.9 |
| Primary source of infection [n (%)] | | | | | |
| Abdomen | 14 | (70.0) | 49 | (61.0) | 0.1 |
| Lung | 3 | (15.0) | 40 | (50.0) | |
| Urinary Tract | 3 | (15.0) | 7 | (9.0) | |
| Others | 1 | (5.0) | 6 | (8.0) | |
| Combination | 1 | (5.0) | 22 | (28.0) | |
| Coexisting conditions [n (%)] | | | | | |
| Chronic kidney disease | 7 | (35.0) | 49 | (61.0) | 0.6 |
| Hypertension | 15 | (75.0) | 59 | (74.0) | |
| Diabetes mellitus | 5 | (25.0) | 28 | (35.0) | |
| Coronary heart disease | 5 | (25.0) | 15 | (19.0) | |
| Cancer | 11 | (55.0) | 41 | (51.0) | |
| | | | | | |
| SOFA sore at admission [median (IQR)] (points) | 13.0 | (12.0 – 14.5) | 12.0 | (9.0 – 14.0) | 0.1 |
| Plasmatic lactate at admission [median (IQR)] (mg/dl) | 37.6 | (23.6 – 75.7) | 33.6 | (20.1 – 58.4) | 0.4 |
| | | | | | |

No data on race or ethnicity was collected. Abbreviations: BMI, Body Mass Index; IQR, inter quartile range; SOFA, Sequential Sepsis-related Organ Failure Assessment

## Supplemental Table S5: Supportive statistics from MG, LPS and TNF titration.

Methylglyoxal Titration

**Dunn's multiple comparisons test with adjusted p Value**

| **0.5 µM** | 1h | | 3h | | 5h |
|---|---|---|---|---|---|
| 0h | 0.6514 | | 0.008 | | 0.0008 |
| | ns | | ** | | *** |
| **1 µM** | 1h | | 3h | | 5h |
| 0h | 0.1922 | | 0.0384 | | 0.0011 |
| | ns | | * | | ** |
| **1.5 µM** | 1h | | 3h | | 5h |
| 0h | 0.4312 | | 0.0042 | | 0.0034 |
| | ns | | ** | | ** |
| **100 µM** | 1h | 2h | 3h | 4h | 5h |
| 0h | 0.1512 | 0.0193 | 0.0284 | 0.0025 | 0.0003 |
| | ns | * | * | ** | *** |
| **200 µM** | 1h | 2h | 3h | 4h | 5h |
| 0h | 0.0764 | 0.011 | 0.0372 | <0.0001 | <0.0001 |
| | ns | * | * | **** | **** |
| **300 µM** | 1h | 2h | 3h | 4h | 5h |
| 0h | 0.8969 | 0.0472 | 0.0157 | <0.0001 | <0.0001 |
| | ns | * | * | **** | **** |
| **400 µM** | 1h | 2h | 3h | 4h | 5h |
| 0h | 0.0392 | 0.0842 | 0.0101 | <0.0001 | <0.0001 |
| | * | ns | * | **** | **** |
| **500 µM** | 1h | 2h | 3h | 4h | 5h |
| 0h | 0.1169 | 0.0645 | 0.1219 | <0.0001 | <0.0001 |
| | ns | ns | ns | **** | **** |

LPS Titration

**Dunn's multiple comparisons test with adjusted p Value**

| **1 µg/ml** | 1h | 3h | 5h |
|---|---|---|---|
| 0h | 0.2868 | 0.0091 | 0.0032 |
| | ns | ** | ** |
| **2 µg/ml** | 1h | 3h | 5h |
| 0h | 0.5431 | 0.0455 | 0.0049 |
| | ns | * | ** |
| **5 µg/ml** | 1h | 3h | 5h |
| 0h | 0.8541 | 0.0482 | 0.0018 |
| | ns | * | ** |

TNF Titration

**Dunn's multiple comparisons test with adjusted p Value**

| **10 µg/ml** | 1h | 3h | 5h |
|---|---|---|---|
| 0h | 0.2992 | 0.0545 | 0.0009 |
| | ns | ns | *** |
| **20 µg/ml** | 1h | 3h | 5h |
| 0h | >0.9999 | 0.8 | 0.0323 |
| | ns | ns | * |
| **30 µg/ml** | 1h | 3h | 5h |
| 0h | >0.9999 | >0.9999 | 0.0269 |
| | ns | ns | * |
| **40 µg/ml** | 1h | 3h | 5h |
| 0h | >0.9999 | 0.2072 | 0.0457 |
| | ns | ns | * |
| **50 µg/ml** | 1h | 3h | 5h |
| 0h | 0.7478 | 0.0175 | 0.008 |
| | ns | * | ** |

Concerning symbols: ns, not significant; * $p \leq 0.05$; ** $p \leq 0.01$; *** $p \leq 0.001$; **** $p \leq 0.0001$

Abbreviations: LPS, lipopolysaccharide; MG, methylglyoxal; TNF, tumor necrosis factor.

Supplementary Information
Schmoch Th. et al., Anserine reduces mortality in experimental sepsis by preventing methylglyoxal induced capillary leakage

**References for Supplementary Information**


1. Brenner T, Fleming T, Uhle F, Silaff S, Schmitt F, Salgado E, u. a. Methylglyoxal as a new biomarker in patients with septic shock: an observational clinical study. Crit Care Lond Engl. 12. Dezember 2014;18(6):683.
2. Nusshag C, Rupp C, Schmitt F, Krautkrämer E, Speer C, Kälble F, u. a. Cell Cycle Biomarkers and Soluble Urokinase-Type Plasminogen Activator Receptor for the Prediction of Sepsis-Induced Acute Kidney Injury Requiring Renal Replacement Therapy: A Prospective, Exploratory Study. Crit Care Med. Dezember 2019;47(12):e999–1007.
3. Bartling B, Zunkel K, Al-Robaiy S, Dehghani F, Simm A. Gene doubling increases glyoxalase 1 expression in RAGE knockout mice. Biochim Biophys Acta BBA - Gen Subj. 1. Januar 2020;1864(1):129438.
4. Singer M, Deutschman CS, Seymour CW, Shankar-Hari M, Annane D, Bauer M, u. a. The Third International Consensus Definitions for Sepsis and Septic Shock (Sepsis-3). JAMA. 23. Februar 2016;315(8):801–10.
5. Levy MM, Fink MP, Marshall JC, Abraham E, Angus D, Cook D, u. a. 2001 SCCM/ESICM/ACCP/ATS/SIS International Sepsis Definitions Conference. Intensive Care Med. April 2003;29(4):530–8.
6. ZO-1 Monoclonal Antibody (ZO1-1A12), Alexa Fluor™ 647 (MA3-39100-A647) [Internet]. [zitiert 25. Februar 2025]. Verfügbar unter: https://www.thermofisher.com/antibody/product/ZO-1-Antibody-clone-ZO1-1A12-Monoclonal/MA3-39100-A647
7. PE Mouse anti-NF-κB p65 (pS529) [Internet]. [zitiert 25. Februar 2025]. Verfügbar unter: https://www.bdbiosciences.com/en-us/products/reagents/flow-cytometry-reagents/research-reagents/single-color-antibodies-ruo/pe-mouse-anti-nf-b-p65-ps529.558423
8. Claudin 5 Monoclonal Antibody (4C3C2), Alexa Fluor™ 488 (352588) [Internet]. [zitiert 25. Februar 2025]. Verfügbar unter: https://www.thermofisher.com/antibody/product/Claudin-5-Antibody-clone-4C3C2-Monoclonal/352588
9. Technology CS. Cell Signaling Technology. [zitiert 25. Februar 2025]. Phospho-c-Jun (Ser73) (D47G9) XP® Rabbit mAb (PE Conjugate). Verfügbar unter: https://www.cellsignal.com/products/antibody-conjugates/phospho-c-jun-ser73-d47g9-xp-rabbit-mab-pe-conjugate/8752?srsltid=AfmBOooDY8uCTal-9LuYgw9YvW5nzZcH_cfITjTMWetTJCRd2I1sV2sW
10. Technology CS. Cell Signaling Technology. [zitiert 25. Februar 2025]. Phospho-Akt (Ser473) (D9E) XP® Rabbit mAb (Alexa Fluor® 647 Conjugate). Verfügbar unter: https://www.cellsignal.com/products/antibody-conjugates/phospho-akt-ser473-d9e-xp-rabbit-mab-alexa-fluor-647-conjugate/4075?srsltid=AfmBOopSUqqZ7nX5VNG-nDj00Wp-vknxuHCShW3xp3032jw694ppdmRc
11. Technology CS. Cell Signaling Technology. [zitiert 25. Februar 2025]. Phospho-p38 MAPK (Thr180/Tyr182) (28B10) Mouse mAb (Alexa Fluor® 488 Conjugate). Verfügbar unter: https://www.cellsignal.com/products/antibody-conjugates/phospho-p38-mapk-thr180-tyr182-28b10-mouse-mab-alexa-fluor-488-conjugate/4551?srsltid=AfmBOorfCRnrrCVcWkOc8I9ziFirwQx-8vgLipTTH0m04Q8F63ML-fUD
12. Technology CS. Cell Signaling Technology. [zitiert 25. Februar 2025]. Phospho-IRF-3 (Ser386) (E7J8G) XP® Rabbit mAb (Alexa Fluor® 647 Conjugate). Verfügbar unter: https://www.cellsignal.com/products/antibody-conjugates/phospho-irf-3-ser386-e7j8g-xp-rabbit-mab-alexa-fluor-647-conjugate/96421?srsltid=AfmBOoqfVJ3DLdjFeHU_2m9mJE-9Gw3OsxJoJ8mPnpMPZ27zQ5LSfTDW
13. Anti-AGE antibody. Rabbit polyclonal (ab23722) | Abcam [Internet]. [zitiert 25. Februar 2025]. Verfügbar unter: https://www.abcam.com/en-us/products/primary-antibodies/age-antibody-ab23722?srsltid=AfmBOor0cXqHdeNJqJXdjz1_j41IpxjBvEEEdN-3i6rOcKsJxOFkSIBZ#tab=datasheet
